# Therapeutic effects of hypoimmunogenic universal human iPSC-derived endothelial cells in a humanized mouse model of peripheral artery disease

**DOI:** 10.1186/s13287-025-04554-5

**Published:** 2025-08-06

**Authors:** Jungju Choi, Nam Gyo Kim, Dasom Kong, Min-Ji Kim, Byeong-Cheol Kang, Daekee Kwon, Da-Hyun Kim, Kyung-Sun Kang

**Affiliations:** 1 Adult Stem Cell Research Center and Research Institute for Veterinary Medicine, College of Veterinary Medicine, Seoul National University, Seoul, 08826 Republic of Korea; 2 Department of Experimental Animal Research, Biomedical Research Institute, Seoul National University Hospital, Seoul, Republic of Korea; 3 Research Institute in Maru Therapeutics, Seoul, 02455 Republic of Korea; 4 Department of Biotechnology, Sungshin Women’s University, Seoul, 01133 Republic of Korea

**Keywords:** Hypoimmunogenic iPSCs, Endothelial cells, Angiogenesis, Humanized mouse, Hindlimb ischemia, Immune response

## Abstract

**Background:**

Peripheral arterial disease (PAD) is a leading cause of limb disability due to ischemia caused by atherosclerotic plaques. Cell-based therapies using endothelial cells (ECs) have shown promise in promoting angiogenesis for PAD, but challenges remain in obtaining sufficient ECs from human tissues. Induced pluripotent stem cells (iPSCs) provide a potential solution, though immune rejection issues arise due to human leukocyte antigen (HLA) mismatches. The depletion of HLA class I and II through gene editing aims to broadly avoid lymphocyte recognition and can be achieved by inactivating β2-microglobulin (B2M) and class II transactivator (CIITA). However, B2M inactivation can lead to a ‘missing self’ killing response by NK cells and macrophages. To overcome this, we proposed universal iPSCs by knocking out B2M and CIITA and over-expressing CD24 to reduce immune rejection.

**Methods:**

Universal iPSCs were tested for their pluripotency and ability to differentiate into ECs. The stimulation of universal iPSC-derived endothelial cells (U-ECs) to T and NK cells was evaluated by activation marker using flow cytometry. We generated a humanized mouse model by intravenously injecting CD34^+^ hematopoietic stem cells isolated from umbilical cord blood into NSG mice. Finally, we induced a humanized PAD mouse model by removing the femoral artery of the left hindlimb. Then we injected U-ECs, demonstrating the therapeutic efficacy of U-ECs.

**Results:**

We generated hypoimmunogenic universal iPSC by knocking out B2M and CIITA, along with over-expressing CD24, and confirmed that their pluripotency was maintained. We demonstrated that U-ECs exhibit functional endothelial properties and reduced immunogenicity, effectively mitigating immune recognition from both adaptive and innate immune responses. U-ECs survived in significantly greater numbers after transplantation and elicited a weaker immune response in humanized mice. Then we induced hindlimb ischemia in humanized mice to establish a humanized PAD model. U-ECs induced effective angiogenic capabilities, leading to significant blood flow restoration in ischemic limbs.

**Conclusions:**

This study demonstrates the feasibility of creating hypoimmunogenic iPSCs and their derivatives that can reduce immune response and function effectively in vivo.

**Supplementary Information:**

The online version contains supplementary material available at 10.1186/s13287-025-04554-5.

## Background

Peripheral arterial disease (PAD) occurs due to atherosclerotic plaque accumulation, which results in ischemia in the lower limbs [1]. PAD remains one of the primary causes of limb disability and loss, which is caused by critical limb ischemia [2]. Currently, more than 200 million people worldwide are affected by PAD, ranging from asymptomatic to severe conditions, and with an aging population, this number is expected to double by 2050 as the disease burden is closely linked to age [3, 4]. Despite the disease's significant impact on quality of life and its high risk of amputation, there are currently limited treatment options available. Surgical and endovascular procedures to restore blood flow to the ischemic limb are effective, but not all PAD patients are suitable. The main treatment approach for patients with critical limb ischemia is revascularization. A potential method to induce revascularization is therapeutic angiogenesis, which aims to stimulate the growth of new blood vessels from existing ones [5, 6]. Recently, several studies in cell therapy reported that cells have the potential to revascularize the ischemic limb [7].

Cell-based therapy using endothelial cells (ECs) has been envisioned as a promising strategy to enhance angiogenesis for the treatment of PAD. ECs have demonstrated the ability to promote tissue regeneration and restore vascular function through angiogenesis, neovascularization, and vascular repair [8–10]. This process results in the formation of new blood vessels and increased branching of existing vessels. However, obtaining human tissues for isolation and cultivating primary ECs from patients is difficult due to time and cost constraints.

Induced pluripotent stem cells (iPSCs) are considerably useful for cellular therapy due to their unlimited capacity for self-renewal and their ability to differentiate into almost all cell types in the human body [11]. Consequently, human iPSCs are attractive cell sources for the clinical-scale preparation of ECs. However, several challenges remain, such as ensuring the efficient survival, engraftment, and long-term functionality of transplanted hiPSC derivatives. Although patient-specific autologous approaches have been proposed [12], this method was constrained by scalability, variability, and quality control. Therefore, the generation of allogeneic and immune-evasive cells is necessary for iPSC-based cell therapy.

Major histocompatibility complexes (MHCs) are responsible for immunogenic recognition. Transplantation of MHC-mismatched iPSC derivatives could induce a potent immune rejection response, in which the recipient’s T cells recognize and reject allogeneic donor cells presenting non-self determinants via MHCs [13]. MHCs are also referred to as human leukocyte antigens (HLAs) in humans. HLA class I molecules, such as HLA-A, -B, and -C, are found on almost all nucleated cells and platelets. They present intracellularly processed peptides to CD8^+^ cytotoxic T cells and enable the elimination of antigen-expressing or virus-infected cells. On the other hand, HLA class II molecules, such as HLA-DP, -DQ, and -DR, are almost exclusively expressed in antigen-presenting cells to present antigens to CD4^+^ helper T cells. Beta-2 microglobulin (B2M) is a component of the HLA class I, and class II transactivator (CIITA) is critical for the production of HLA class II [14]. The depletion of HLA class I and II through gene editing aims to broadly avoid lymphocyte recognition and can be achieved by inactivating B2M and CIITA [15]. However, B2M inactivation can lead to a ‘missing self’ killing response by natural killer (NK) cells and macrophages, both of which recognize HLA class I deficiency [16, 17].

To prevent cell lysis by NK cells, overexpression of CD24, a “don’t-eat-me” signal, has been designed to protect the engineered cells. Tumor-expressed CD24 promoted immune evasion by interacting with the inhibitory receptor sialic-acid-binding Ig-like lectin 10 (Siglec-10), which is expressed by M2-like tumor-associated macrophages (TAMs) [18]. In a preclinical model of CD24^+^ solid tumors, blocking the CD24-Siglec-10 interaction with an anti-CD24 monoclonal antibody (mAb) led to increased TAM-associated phagocytosis in vitro, as well as TAM-dependent tumor growth reduction and enhanced survival in vivo [18]. CD24 might play a crucial role in the anti-phagocytic signaling process, as mantle cell lymphoma and chronic lymphocytic leukemia represent a subset of B-cell malignancies with a significantly hostile tumor microenvironment (TME) with M2-like TAMs [19–25].

Historically, mouse models have served as useful in vivo tools for preclinical studies of various human therapies. Nevertheless, because of obvious physiological differences between mice and humans, the results from these models do not always accurately predict human outcomes [26]. To create more appropriate in vivo models, mice have been humanized to mimic complex human physiological processes by transplanting functional human tissues into immunodeficient mice [27]. Notably, recent advancements in technology have improved the creation of humanized mice with human immune systems, allowing for the long-term engraftment of human hematopoietic stem cells and the generation of various hematopoietic lineages, including B and T cells [27, 28].

According to our previous work, we produced multiplex gene-edited (B2M^−/−^ CIITA^−/−^ TRA^−/−^ PDCD1^−/−^ CTLA4^−/−^ CD19-CAR^a/e^ CD24^a/e^) iPSCs/NK cells through the integration of cellular reprogramming, gene editing, and differentiation technologies [29]. We also demonstrated the efficacy of CD19 CAR-iNK cells in targeting pericytes within the glioblastoma tumor microenvironment [30]. Here, based on these previous findings, we combined these engineering approaches and designed B2M^−/−^CIITA^−/−^CD24^a/e^ human-engineered universal iPSCs (U-iPSCs). We evaluated the therapeutic efficacy of our U-iPSC-derived endothelial cells (U-ECs) in promoting angiogenesis using a hindlimb ischemia model in humanized mice. U-ECs effectively inhibit allogeneic adaptive and innate immune activation. Compared to wild-type iPSC-derived endothelial cells (WT-ECs), U-ECs survived longer, leading to a significant recovery of blood flow in the humanized PAD mouse model. Therefore, our U-ECs could be a valuable tool for universal cell-based therapies.

## Materials and methods

### Cell culture

This study was carried out following the approval under the project title of “Development of immune-modulated 3D tissue complex and regeneration technology for the incurable liver disease” by the Seoul National University Institutional Review Board (IRB No. 2212/001–014, 29th December 2022). The human iPSC lines WT and U-iPSCs were obtained from the MARU THERAPEUTICS for the generation of endothelial cells under the project title of “Development of anti-cancer immune cell therapy and antiviral cell therapy derived from human-induced pluripotent stem cells” (IRB NO. P01-202110–31-009, 20th October 2021). The genetic engineering of iPSCs was conducted as described in our previous study [29]. These iPSCs were maintained in StemFLEX medium (Gibco, USA) on dishes coated with growth-factor-reduced(GFR) Matrigel (Corning, USA). The iPSC cells were passaged every 3 ~ 4 days using ReLeSR reagent (STEMCELL Technologies, Canada). Human umbilical vein endothelial cells (HUVECs) purchased from ATCC (USA) and were maintained in endothelial growth medium-2 (EGM-2, Lonza, Switzerland) supplemented with 10% fetal bovine serum (FBS, Gibco). Cells were maintained at 37ºC in a humidified incubator with 5% CO2.

### Whole genome sequencing

This library prep set is optimized to convert 5–400 ng genomic DNA into a customized library and uses advanced Adapter Ligation technology and High-fidelity PCR Enzymes to significantly increase library yield and conversion rate. Library preparation was performed with MGIEasy FS DNA library prep kit (MGI) according to the manufacturer's instructions. Briefly, after size-selection of fragmented gDNA using AMPure XP magnetic beads, the fragmented gDNA was end-repaired and a-tailed at 37 °C for 30 min, and 65 °C for 15 min. Indexing adapter was ligated to the ends of the DNA fragments at 23 °C for 30 min. After cleanup of adapter-ligated DNA, PCR was performed to enrich those DNA fragments that have adapter molecules. Thermocycler conditions were as follows: 95 °C for 3 min, 8 cycles of 98 °C for 20 s, 60 °C for 15 s, and 72 °C for 30 s, with a final extension at 72 °C for 10 min. The double stranded library was quantified using QauntiFluor ONE dsDNA System (Promega). The library was circularized at 37 °C for 30 min, and then digested at 37 °C for 30 min, followed by cleanup of circularization product. To make DNA nanoball (DNB), the library was incubated at 30 °C for 10 min using DNB enzyme. Finally, Library was quantified by QauntiFluor ssDNA System (Promega). Sequencing of the prepared DNB was conducted on the MGIseq system (MGI) with 150 bp paired-end reads.

### Quantitative real-time polymerase chain reaction (qRT-PCR)

Prepared RNA of cell lines was extracted from 1 × 10^6^ cells with Trizol reagent (Invitrogen, USA). Complementary DNA synthesis was conducted using a Superscript III First-Strand synthesis system kit (Invitrogen) according to the manufacturer’s instructions. qRT-PCR analysis was performed with SYBR Green PCR Mix (Applied Biosystems, USA) on a 7500 Real-Time PCR system (Applied Biosystems). The relative mRNA expression of each target gene was quantified by the 2^(ΔΔ threshold cycle)^ (2^−ΔΔCT^) method. The level of target mRNA was normalized relative to the amount of GAPDH as a housekeeping control. The primers used in this study are shown in Supplementary Table 1.

### Flow cytometry analysis

Cells were dissociated with TrypLE™ and dissociated single cells were stained with fluorescence conjugated antibodies. MACSQuant Analyzer 10 (Miltenyi Biotec) was used for cell analysis. Manual gating for flow cytometry analysis was performed by FlowJo v.10 (BD Biosciences) and MACSQuantify (Miltenyi Biotec).

### Pluripotency immunofluorescence

Cells on coverslips were fixed in 4% paraformaldehyde for 20 min at room temperature. After three washes in PBS, cells were blocked with 5% normal goat serum containing 0.1% Triton X-100 at room temperature for 1 h. Cells were incubated with primary antibodies at 4 °C overnight (Supplementary Table 2), washed three times with PBS and incubated with a secondary antibody for 1 h at room temperature: Alexa Fluor 488-labeled (A11008, A11001, Invitrogen, USA), 594-labeled (A11012, A11005, Invitrogen). Then, the nuclei were stained with DAPI (Invitrogen) for 10 min at room temperature. Finally, the sections were washed in PBS and mounted with a mounting solution (Dako, Denmark). Confocal images were captured by a confocal microscope (Eclipse TE200, Nikon, Japan) with the EZ-C1 3.8 program.

### Endothelial cells differentiation from iPSCs

To differentiate WT-iPSCs and U-iPSCs into ECs, we modified the previous protocol [31]. Briefly, iPSCs were dissociated with TrypLE™ (Gibco) and seeded in StemFLEX medium with 10μM ROCK inhibitor Y-27632 (Tocris, UK) on plates coated with 1:40 GFR Matrigel. After 24 h, the medium was replaced with 1:1 mixture of DMEM/F-12 (Gibco) and Neurobasal media (Gibco) supplemented with 1X N2 (Gibco), 1X B27(Gibco), 1X GlutaMAX(Gibco), 8μM CHIR99021 (Peprotech, USA) and 25 ng/mL BMP4 (Peprotech). After 3 days, the medium was replaced with StemPro-34 SFM medium (Gibco) supplemented with 200 ng/mL VEGF (Biolegend, USA) and 2 µM forskolin (Stemcell Technologies). On day 6, the cells were dissociated with TrypLE™ and went through magnetic-activated cell sorting (MACS) purification according to the manufacturer’s protocol using CD31 MicroBead Kit (130–091-935, Miltenyi Biotec, Germany) for positive selection. After MACS-mediated selection, the CD31-expressing ECs were seeded in EGM-2 medium with 10% FBS, 10 µM SB431542 (peprotech), 50 ng/mL VEGF on fibronectin (Corning)-coated plates.

### Enzyme linked immunosorbent assay (ELISA)

The secretion of VEGF was quantified using the human VEGF Quantikine ELISA Kit (DVE00, R&D Systems, USA). The secretion of endothelin-1, PGI2 and nitric oxide was quantified using Endothelin-1 Quantikine ELISA Kit (DET100, R&D Systems), PGI2 ELISA kit (FY-EU1112, FeiyueBio, China) and NO Plus Detection Kit (21,023, iNtRON, Republic of Korea), respectively. The culture supernatant was collected and centrifuged at 2000 rpm for 5 min to eliminate cell debris.

### *Tube formation *in vitro

To measure the angiogenesis of the iPSC-ECs, we conducted tube formation experiments. Seventy-five microliters of GFR Matrigel per well was added to 96-well plate and incubated for 1 h at 37 °C. 2 × 10^4^ ECs per well were then seeded onto the matrix and cultured in EGM-2 with 10% FBS for 18 h at 37 °C and 5% CO2. HUVEC served as the positive control. The resulting tubes were stained with 6 μM Calcein AM (Invitrogen) at 37 °C for 30 min.

### AcLDL uptake

Endothelial cells were seeded onto 24-well plates (corning). Cells were added 8 μg/mL of Dil-AcLDL (Invtirogen) and then incubated at 37 °C for 4 h. The cells were fixed with 4% paraformaldehyde for 20 min and then washed with PBS. Then, the nuclei were stained with DAPI (Invitrogen) for 10 min at room temperature.

### Isolation of T cells and NK cells from human umbilical cord blood

Human umbilical cord blood (hUCB) was provided by the Seoul Metropolitan Government Public Cord Blood Bank (Allcord, Korea) under the project title of “Development of immune-modulated 3D tissue complex and regeneration technology for the incurable liver disease” approved by the Seoul National University Institutional Review Board (IRB No. E2307/001–006, 4th July 2023). Mononuclear cells (MNCs) were separated by density-gradient centrifugation with Ficoll-Paque plus (Cytiva, USA). MNCs were conjugated with human CD3 microbeads (130–050-101, Miltenyi Biotec), human CD4 microbeads (130–045-101, Miltenyi Biotec), and human CD8 microbeads (130–045-201, Miltenyi Biotec). Positive selection was performed with LS columns (Miltenyi Biotec) according to the manufacturer’s protocol. NK cells were isolated by negative selection with NK cell isolation Kit (130–092-657, Miltenyi Biotec) from MNCs.

### CFSE-labeled T cell proliferation assay

CD3 positive T cells were labeled with CellTrace CFSE (C34554, Invitrogen) according to the manufacturer’s instructions. WT and U-ECs, used as target cells, were seeded in 24-well plates at 1 × 10^4^ cells per well. CFSE-labeled T cells were cocultured with ECs in RPMI supplemented with 10% FBS in the presence of 100 U/ml IL-2 for 6 days. On day 6, cocultured cells were harvested and then stained with anti-hCD3-APC (344,812, Biolegend) for 20 min on ice. After staining, CFSE intensity was analyzed by flow cytometry.

### T cell activation assay

5 × 10^5^ iPSC-derived ECs were seeded onto 24-well plates. CD4 and CD8 positive T cells were isolated from hUCB and 5 × 10^5^ T cells were added to the endothelial cell monolayers at a 1:1 ratio of RPMI-1640 and EGM-2 medium supplemented with 10% fetal bovine serum (FBS) and 100 IU/mL IL-2. As a negative control, T cells were cultured in the same medium without endothelial cells. After 48 h, the supernatant was collected and T cell activation was evaluated by measuring the expression of FITC anti-human HLA-DR (560,944, BD) activation marker, using flow cytometry.

### NK cell degranulation assay

To assess the degranulation of NK cells against ECs, isolated NK cells (5 × 10^4^ cells) from hUCB were co-cultured with ECs (5 × 10^4^ cells) in ultra-low attachment 96-well plates (corning). After 8 h of co-incubation, cells were washed and stained with anti-hCD107a-APC (560,664, BD) for 20 min on ice. The cells were analyzed using flow cytometry. NK cells cultured without target cells were used as negative control, while ones treated with 50 ng/mL PMA (phorbol 12-myristate-13-acetate) (Biogems, USA) and 1 μg/mL ionomycin (Biogems) were used as positive control.

### LDH assay

Target cells (ECs) and effector cells (NK cells) at various indicated effector/target ratios (3:1, 1:1, and 1:3) were co-incubated with 200 μL NK cell medium in ultra-low attachment 96-well plates. After 20 h of incubation, the LDH assay (11644793001, LDH cytotoxicity kit, Roche, Switzerland) was performed by following manufacturer’s instructions. NK cytotoxicity was calculated as NK cell cytotoxicity (%) = [(NK and ECs co-culture LDH release − spontaneous LDH release)/(maximum LDH release − spontaneous LDH release)] × 100. NK cell medium was used as background control. ECs cultured alone were used as controls for spontaneous LDH release. Lysed ECs at endpoint were used as maximal LDH release.

### Annexin V/7-aad assay

To assess immune cell-mediated apoptosis, CD3-positive T cells and NK cells were co-incubated with ECs in 96-well U bottom plates. The medium consisted of a 1:1 mixture of RPMI 1640 and EGM-2, supplemented with 10% FBS and 100 U/mL IL-2. As a negative control, ECs were cultured in the same medium without immune cells. After 24 h, cells were harvested and stained using the PE Annexin V Apoptosis Detection Kit I (559,763, BD), followed by analysis with flow cytometry. Manual gating for flow cytometry analysis was performed by FlowJo v.10 (BD).

### Establishment of humanized mouse model

All experimental procedures were approved by the Institutional Animal Care and Use Committee of Seoul National University (IACUC SNU-230621–4-2). NOD.C-Prkdc^scid^IL2rg^tmlWjl^/Sz (NSG) mice were obtained from GEM BIOSCIENCES (Korea) and housed in semi-SPF barrier facility in a 12 h dark/light cycle, temperature- and humidity-controlled environment. Sterile cages, bedding, food, and water were given. The sample size was determined based on the specific requirements of each experiment. Randomization was used to allocate experimental units within each cage. The group allocation was carried out by the researcher who performed the randomization. Animals were anesthetized using isoflurane at 2–3% in oxygen. Humane endpoints were established to minimize animal suffering and distress during the study. A weight loss of more than 20% of the animal's body weight was considered an indicator of significant distress or health decline. Mice showing signs of extreme lethargy, inability to move, or lack of responsiveness to stimuli were monitored closely. Any signs of visible pain or distress, such as labored breathing, hunched posture, excessive vocalization, or abnormal grooming, were considered serious and required intervention. Any drastic changes in behavior, such as aggressive behavior, disorientation, or seizures, were closely monitored. Any signs of infection or significant wound healing problems were grounds for euthanasia. At the end of the experimental protocol or in case of reaching the humane endpoint, animals were euthanized using CO2 asphyxiation. The work has been reported in line with the ARRIVE guidelines 2.0.

For humanization, 6 week-old NSG male mice were intraperitoneally injected with busulfan (25 mg/kg; MilliporeSigma, USA) twice, once two days and again one day prior to transplantation, to achieve bone marrow depletion. 1 × 10^5^ cells of human CD34^**+**^ hematopoietic stem cells (HSCs) were subsequently transplanted by tail vein intravenous injection. HSCs were sorted from hUCB-derived MNCs by using CD34 microbeads (130–046-703, Miltenyi Biotec). Although it has been reported that fresh cord blood-derived HSCs generally show higher engraftment efficiency than cryopreserved cells [32], we conducted our experiments using donated human umbilical cord, which limited the feasibility of obtaining large quantities of fresh cord blood at once. Therefore, for experimental practicality, CD34^**+**^ HSCs were isolated from multiple cord blood collected over time and cryopreserved. To secure sufficient cell numbers and reduce donor variations, these cells were pooled before injection. Six weeks after transplantation of CD34^**+**^ HSCs, peripheral blood was collected every other week. Blood collection was performed via the orbital sinus under anesthesia using isoflurane. To remove red blood cell, peripheral blood was treated with ACK (Ammonium-Chloride-Potassium) lysing buffer (Gibco). Cells were stained in the dark for 30 min on ice using the following antibodies: anti-hCD45-FITC (555,482, BD), anti-hCD24-PE (560,991, BD), anti-hCD3-APC (344,812, Biolegend) and anti-hCD56-PE (561,903, BD). After staining, the cells were analyzed using a flow cytometry assay. Established humanized mice were further used for cell transplantation.

### In vivo* bioluminescent imaging*

Humanized NSG mice used for bioluminescent imaging were maintained in a semi-SPF facility, where a moderate level of mortality was observed due to immune dysfunction [33]. For xenograft, WT-ECs and U-ECs were embedded in Matrigel (Corning) solution and transplanted subcutaneously into the left flank of mice. D-Luciferin Potassium (122799, Revvity, USA) dissolved in sterile PBS was injected intravenously into humanized mice for bioluminescent imaging. Animals were imaged using the in vivo imaging system (IVIS) (PerkinElmer, USA) and mice were divided into two groups; Group 1: WT-ECs and Group 2: U-ECs. Bioluminescent imaging was monitored by IVIS every 3–4 days until 14 days post-transplantation. Total flux (photons/sec) was calculated from data of emitted photons from the regions of interest using the Living image software (PerkinElmer).

### Histological analysis

For tissue staining, mice were sacrificed. Specimens were fixed in 4% paraformaldehyde overnight at 4 °C. After washing with PBS more than three times, the specimens were pooled with 30% sucrose solution until completely submerged at the bottom, then embedded in a mixture of 10% gelatin and 30% sucrose solution, and then frozen at − 80°C. After the specimens were cryo-sectioned into 20 μm thick, the sections were washed in PBS at 37 °C for 10 min. For hematoxylin and eosin (H&E) staining, sections were incubated in hematoxylin solution for 7 min and washed with 0.5% HCl in ethanol, followed by counterstaining with eosin for 6 min. After incubating with an increasing sequential ethanol series, the samples were mounted with Canada Balsam in xylene. Samples were visualized with an Olympus microscope and ProgRes CapturePro software (Olympus, Japan). For picrosirius red staining, the sections were incubated in 0.1% picrosirius red solution for 1 h at room temperature. After staining, the sections were washed in 1% acetic acid for 3 min. To quantify the fibrotic area, mean fluorescence intensity (MFI) was measured in the segmented regions using ImageJ (National Institutes of Health, USA). Verhoeff–Van Gieson staining was accomplished using Elastic Stain Kit (ab150667, Abcam, UK) according to the manufacturer’s manual.

### Induction of the hindlimb ischemia model

All the animal experiments were carried out in accordance with the approval by the Seoul National University Hospital Biomedical Research Institute (IACUC No. 24–0036-S1A0). Humanized mice used for the hindlimb ischemia model were housed in a fully SPF-compliant facility, and no mouse death occurred during the experimental period. After eight-weeks CD34^**+**^ HSCs injection, humanized mice were used for inducing the hindlimb ischemia model. After the mice were anesthetized, the femoral artery of the left hindlimb was ligated and excised. Before closing the incision, 1 × 10^6^ cells of ECs were injected into the quadriceps muscle divided into 5 places. HUVECs were used as a control group and PBS was used as a negative control group. The order of treatments was randomized to prevent any systematic bias from treatment sequences.

### Tunnel assay

The cryo-sections were performed the TdT reaction using the Click-iT™ Plus TUNEL Assay Kits for In Situ Apoptosis Detection (C10618, Invitrogen), according to the manufacturer’s instructions. The number of cells in specific areas was manually counted by using ImageJ.

### Elispot assay

For uni-directional Elispot assays, recipient splenocytes were isolated from the spleen 7 days after cell injection and used as responder cells. Donor cells were used as stimulator cells. A total of 5 × 10^5^ stimulator cells were incubated with 5 × 10^5^ recipient responder splenocytes for 24 h and IFN-γ (EL285, BD), IL-2 (EL202, BD), and IL-4 (EL204, BD) spot frequencies were enumerated using an Elispot plate reader.

### Cytokine secretion assay

Serum samples from the mice were collected to analyze systemic inflammation and serum chemistry. Cytokine concentrations in the serum were measured using the human inflammatory cytokine cytometric bead array (CBA) Kit (551811, BD), according to the manufacturer’s instructions. The results were generated using FlowJo v.10.

### Statistical analysis

Statistical analysis was performed using GraphPad Prism version 10.1.2. All values were reported using mean ± standard deviation (SD). The value of p < 0.05 was considered as significant. Statistical significance was determined by unpaired, two-tailed t-test or one-way ANOVA for multiple comparisons. The value of *p* < 0.05 was considered significant (^*^p < 0.05; ^**^p < 0.01; ^***^p < 0.001). The data shown represent at least three independent experiments. The normality of the data was assessed using the Shapiro–Wilk test or Q-Q plots.

## Results

### Generation of hypoimmunogenic iPSCs by CRISPR-Cas9 genome editing

**Fig. 1:**
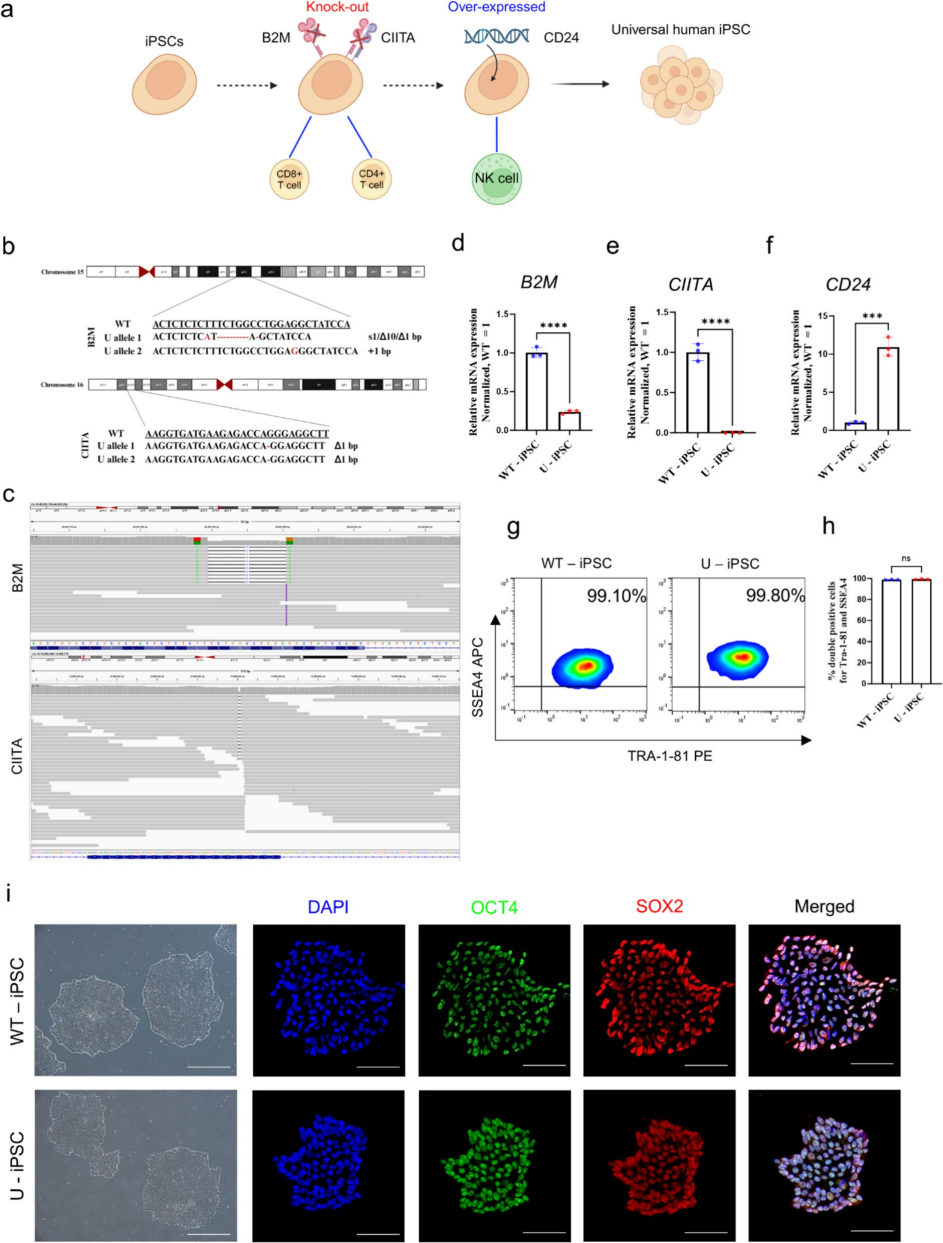
Characterization of hypoimmunogenic iPSCs. **a** Schematic illustration of producing hypoimmunogenic iPSCs by CRISPR-Cas9. **b** Insertions and depletions occurred biallelically in the coding sequences of the U-iPSCs. (c) Read alignments of B2M (chr15: 45,003,788–45,003,801) and CIITA (chr16: 10,989,601) regions area was visualized using the WGS Integrative Genomics Viewer (IGV). The genomic coordinate is hg19. **d**–**f** qRT-PCR of B2M, CIITA, and CD24 in WT and U-iPSCs. Each group n = 3, mean ± SD. ***p < 0.001, ****p < 0.0001 versus WT-iPSCs. **g** Representative flow cytometry analysis of pluripotency markers TRA-1–81 and SSEA4 expression in WT and U-iPSCs. **h** Quantification of the pluripotency in flow cytometry analysis of WT and U-iPSCs (n = 3), mean ± SD, ns; not statistically significant versus WT-iPSCs. **i** Observation of undifferentiated morphology in WT and U-iPSCs using a phase contrast microscope. Representative confocal images of pluripotency (OCT4: green, SOX2: red, DAPI: blue). Scale bars, 50 μm. Statistical differences between the groups were determined by unpaired, two-tailed student’s *t*-test

**Fig. 2:**
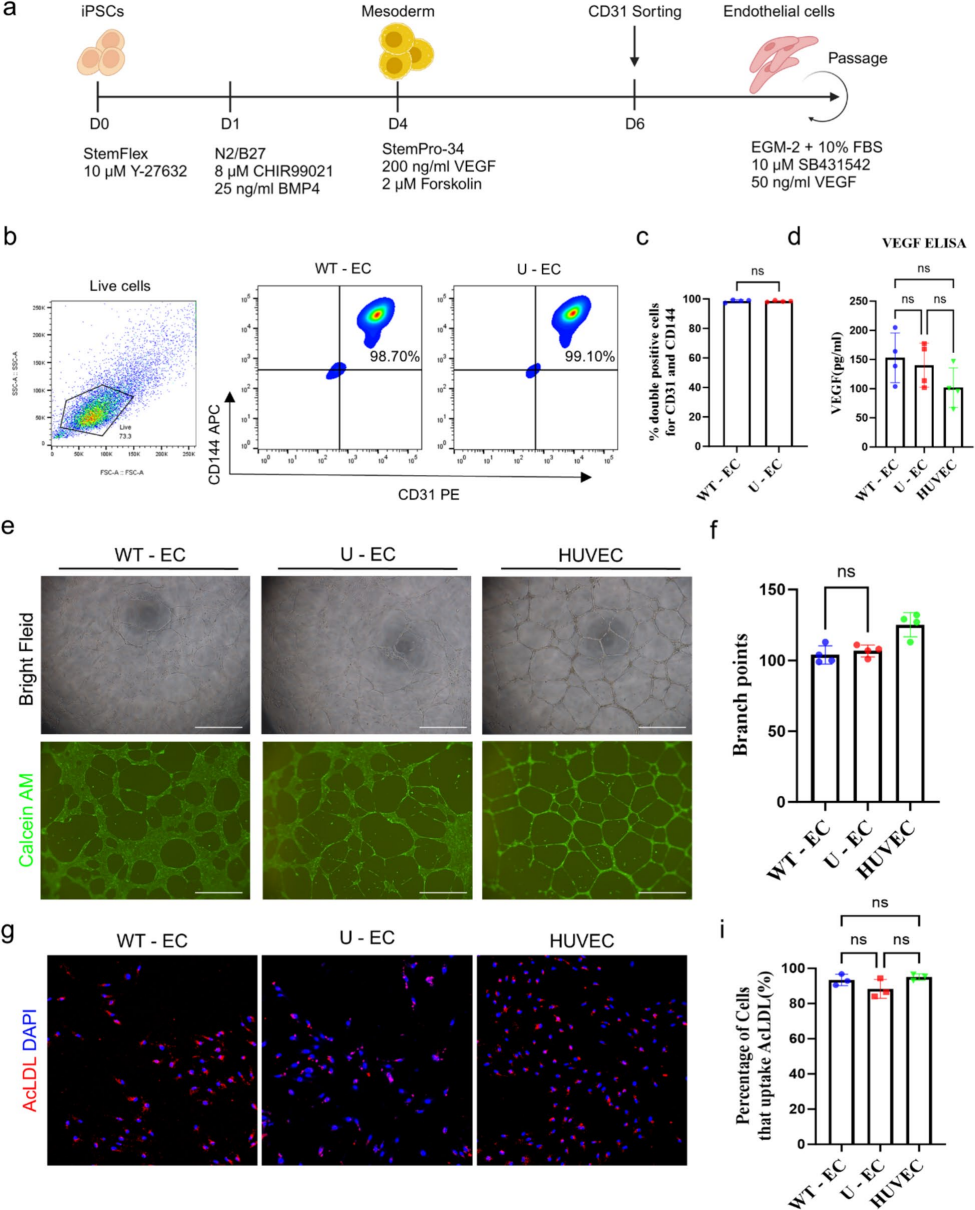
Differentiation of U-iPSCs into endothelial cells. **a** Schematic diagram for the experimental protocol for endothelial cell differentiation. **b**-**c** Representative flow cytometry analysis **b** and quantification **c** of endothelial cell marker expression, CD31 and CD144, on iPSC-ECs differentiated from WT and U-iPSCs. Gated on WT and U-ECs. n = 3, mean ± SD, ns; not statistically significant versus WT-ECs. **d** VEGF secretion levels in the supernatants in ECs as determined by ELISA. n = 4, mean ± SD. ns; not statistically significant. **e** Representative phase-contrast images (top) and fluorescence images (bottom) of tubular structures 24 h after plating on Matrigel. Scale bars, 1 mm **f** Quantification of branching points formed during *in vitro* tubulogenesis. n = 4, mean ± SD. ns; not statistically significant. **g** Uptake of fluorescently labeled AcLDL (red) in the cytoplasm of ECs. Cell nuclei were stained with DAPI (blue). Scale bars, 50 μm **i** Quantification the percentage of ECs that uptake AcLDL. n = 3, mean ± SD. ns; not statistically significant. Statistical differences between the groups were determined by unpaired student’s *t*-test **c** and ordinary one-way ANOVA with post-hoc Tukey test **d**, **f**, **i**

As a previous study described [29], we designed the human-engineered universal iPSCs (U-iPSCs) using the CRISPR-Cas9 gene editing process to establish hypoimmunogenic hiPSCs (Fig. 1A). First, we reduced the immunogenicity of hiPSCs by inactivating the major histocompatibility complex (MHC) class I and II genes to evade T cells. B2M is a structural component of MHC class I, and CIITA is the master regulator of MHC class II molecules. Knockout of B2M and CIITA by CRISPR-Cas9 was confirmed by whole genome sequencing (WGS) of the hiPSCs (Fig. 1B). Read alignments of B2M (chr15: 45,003,788–45,003,801) and CIITA (chr16: 10,989,601) regions area was visualized using the WGS Integrative Genomics Viewer (IGV) (Fig. 2C). We also confirmed the knockout of B2M and CIITA in the U-iPSCs via quantitative RT-PCR (qRT-PCR) (Fig. 1D and E). As the MHC class knockout renders cells susceptible to NK cell attack, we over-expressed the ‘don’t eat me signal’ CD24. We verified the over-expression of CD24 compared to WT-iPSCs via qRT-PCR (Fig. 1F). To ensure that the hiPSCs maintained high quality after CRISPR-Cas9 gene editing, we evaluated the pluripotency of U-iPSCs. The pluripotency was validated by the expression of TRA-1–60 and TRA-1–81 markers through flow cytometry. The results showed that the double positive expressions of pluripotency markers were WT-iPSCs (99.10%) and U-iPSCs (99.80%) (Fig. G and H). WT and U-iPSCs were found to maintain a typical undifferentiated cell morphology. Immunostaining experiments revealed that WT-iPSCs and U-iPSCs had a uniform nuclear expression of typical pluripotent transcription factors, including OCT4 and SOX2 (Fig. 1I). These data suggest that our human-engineered universal iPSCs successfully underwent B2M and CIITA knockout, overexpressed CD24, and retained their pluripotency.

### Differentiation of U-iPSCs into endothelial cells exhibiting the functional characteristics of angiogenesis

To demonstrate the potential application of U-iPSCs, we attempted to differentiate the U-iPSCs into functional endothelial cells (ECs) with previous differentiation protocol [31] (Fig. 2A). The hiPSCs were dissociated into single cells and seeded on growth factor reduced Matrigel in StemFLEX medium. First, hiPSCs were directed to differentiate into the mesodermal stage over a period of 3 days and subsequently replaced with EC inducing medium for another 2 days. To purify ECs, we used magnetic-activated cell sorting (MACS) targeting the endothelial cell surface marker CD31. After CD31^+^ cells were magnetically sorted, the ECs were expanded in EGM-2 medium supplemented with 10% FBS, 10μM SB431542 and 50ng/ml VEGF. To verify successful differentiation, the hiPSC-derived ECs were evaluated by flow cytometry for expression of endothelial markers, such as CD31 and CD144. Both WT-ECs and U-ECs expressed CD31 and CD144 markers at levels exceeding 99% (Fig. 2B and C). To analyze the vascular function of hiPSC-derived ECs, an in vitro tube formation assay was first performed to assess their angiogenic potential, using human umbilical vein endothelial cells (HUVECs) as a control. We conducted an enzyme-linked immunosorbent assay (ELISA) to detect and quantify the concentration of vascular endothelial growth factor (VEGF) in WT-ECs, U-ECs, and HUVECs (Fig. 2D). The formation of vascular network structures by WT-ECs, U-ECs, and HUVEC was observed within 18 h (Fig. 2E). The vascular networks formed by both WT-ECs and U-ECs were less extensive than those formed by HUVECs (Fig. 2F). Acetylated low-density lipoprotein (AcLDL) uptake is an important function of endothelial cells, particularly in their role in vascular health. Both WT-ECs and U-ECs exhibited a similar AcLDL uptake function compared to HUVECs (Fig. G and I). Given the pivotal roles of nitric oxide, PGI2, and endothelin-1 in regulating vascular tone [34–36], we measured their secretion levels in WT-ECs and U-ECs after inflammatory stimulation with 100 ng/mL of lipopolysaccharide (LPS) for 6 h. After 24-h maintenance period, the conditioned medium was collected from each group for ELISA analysis. We found that there was no statistical significance in the levels of nitric oxide, PGI2 and endothelin-1 between WT-ECs and U-ECs (Fig. S1A, S1B and S1C), indicating that both ECs retain comparable functional capacities to release vasoactive mediators under inflammatory conditions. To further assess the paracrine effects of these ECs on vascular smooth muscle cells (VSMCs), the contractile VSMCs were differentiated from the same iPSC line [37]. These induced VSMCs (iVSMCs) were cultured for 2 days in conditioned medium obtained from LPS-treated WT-ECs and U-ECs. As a result, qPCR analysis revealed a significant upregulation of contractile marker genes, such as *MYH11, SM22α, αSMA*, and *Calponin-1* in iVSMCs exposed to conditioned media from both WT-ECs and U-ECs, compared to those maintained in standard culture medium (Fig. S1D). In contrast, expression of the synthetic phenotype-associated gene *MMP-1* was downregulated, indicating a phenotypic shift toward a contractile state. These results demonstrated that iPSC-derived ECs are functionally capable of producing vasoactive mediators and modulating VSMC phenotypes in a paracrine manner. Finally, to evaluate the proliferation capacity of ECs upon passaging, the cumulative population doubling level (CPDL) analysis was conducted. Although the proliferation rates declined with increasing passage numbers in both cell lines, there was no significant difference in proliferative capacity between the WT-ECsqk and U-ECs (Fig. S2A). We also demonstrated how prolonged culture affects the proliferative phenotype of ECs derived from each iPSC line, we analyzed the expression of key cell cycle-related genes across different passages (p1 and p4). We observed that the decreased expression of genes involved in cell cycle progression, including *CCNA2, CCNB1, CDK1*, and *CDK2* with increasing passage number in both WT-ECs and U-ECs (Fig. S2B). This tendency indicated a progressive decline in cell cycle activity over time, which was consistent with CPDL data. However, there was no statistically significant difference in the expression of these genes between WT-ECs and U-ECs at either passage. Taken together, genetic modification introduced into universal iPSCs did not influence the vascular functions as well as proliferative capacity of differentiated ECs.

### Stimulation of T cells using iPSC-derived endothelial cells

**Fig. 3:**
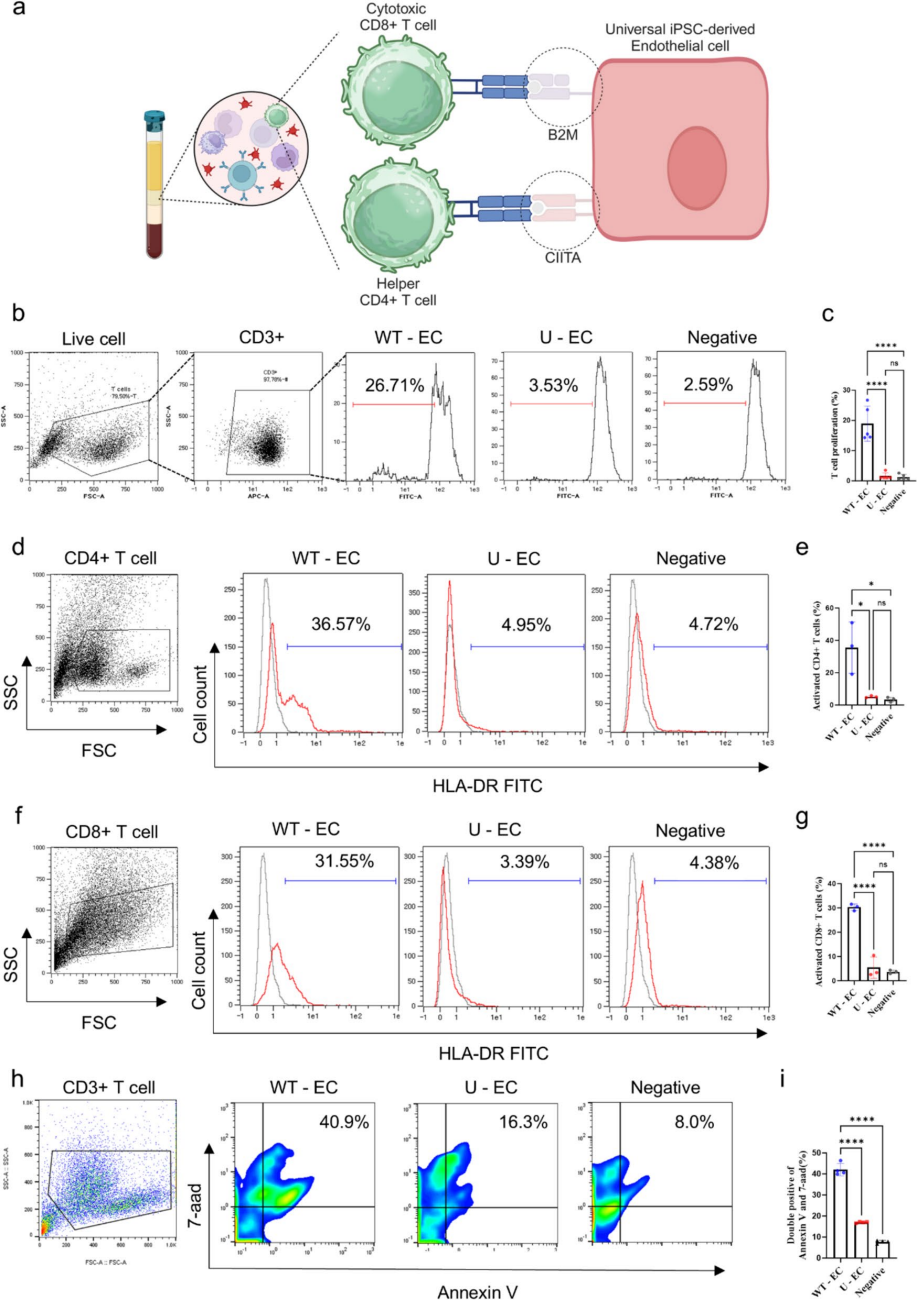
In vitro T cell activations by iPSC-ECs. **a** Schematic diagram of the strategy for evading CD4^+^ and CD8^+^ T cells by knocking out B2M and CIITA in U-ECs. **b** Representative flow cytometry analysis of T cells expression, gated by forward and site scattering plots and CD3-postive plots. T cell proliferation assay with flow cytometry analysis: the percentage of proliferating T cells were plotted by gating of the reduced CFSE fluorescence. T cells cultured alone were used as negative controls. **c** Quantification the percentage of T cell proliferation in flow cytometry analysis. n = 5, mean ± SD, ****p < 0.0001, ns; not statistically significant. **d**-**g** Flow cytometry analysis of HLA-DR on CD4^+^ (d) and CD8^+^ (f) T cells: HLA-DR^+^ cells served as a T cel activation marker. Histogram of percentage of activated CD4^+^ (e) and CD8^+^ (g) T cells. T cells cultured alone were used as negative controls. Each group n = 3, mean ± SD, *p < 0.05, ****p < 0.0001, ns; not statistically significant. **h** Representative flow cytometry analysis of apoptosis in ECs co-cultured with T cells, using annexin V and 7-AAD double-positive expression. **i** Quantification of apoptotic cells. Each group n = 4, mean ± SD, ****p < 0.0001. Statistical differences between the groups were determined by ordinary one-way ANOVA with post-hoc Tukey test

HLAs are responsible for immunogenic recognition. HLA-mismatched iPSC derivatives could induce a potent immune rejection response, where the recipient’s T cells recognize and reject allogeneic donor cells presenting non-self determinants via HLAs. T cells would not be stimulated to proliferate when they encounter HLA knockout cells (Fig. 3A). To investigate whether HLA knockout can reduce the immune stimulation to T cells, a T cell proliferation assay was conducted to assess the immune compatibility of the U-ECs. CD3^+^ T cells were isolated from UCB-MNCs using MACS sorting and labeled with CFSE (Fig. S3A). The percentage of proliferating CD3^+^ T cells in the WT-ECs group was the highest (26.71%) among experimental groups, followed by the U-EC group (3.53%) and the negative control group (2.59%) (Fig. 3B and C).

HLA class I molecules present intracellularly processed peptides to CD8^+^ cytotoxic T cells, while HLA class II molecules are almost exclusively expressed on antigen-presenting cells to present antigens to CD4^+^ helper T cells. We first seeded the iPSC-derived ECs and then isolated CD4^+^ and CD8^+^ T cells from UCB-mononuclear cells (Fig. S3B and S3C) to co-culture them for 6 days. After 6 days, the media were collected, and flow cytometry was performed to assess the expression of T cell activation markers, such as HLA-DR. We observed that both CD4^+^ and CD8^+^ T cells were activated when co-cultured with WT-ECs, while their activation percentage was significantly lower, similar to the negative control, when co-cultured with U-ECs (Fig. 3D-G). Quantitative flow cytometry analysis revealed a significant increase of apoptosis in WT-ECs co-cultured with CD3^+^ T cells (Fig. 3H and I). These results indicate that U-ECs inhibited the proliferation of CD3^+^ T cells and the activation of CD8^+^ cytotoxic T cells and CD4^+^ helper T cells.

### Reduced NK cell response to U-ECs

**Fig. 4:**
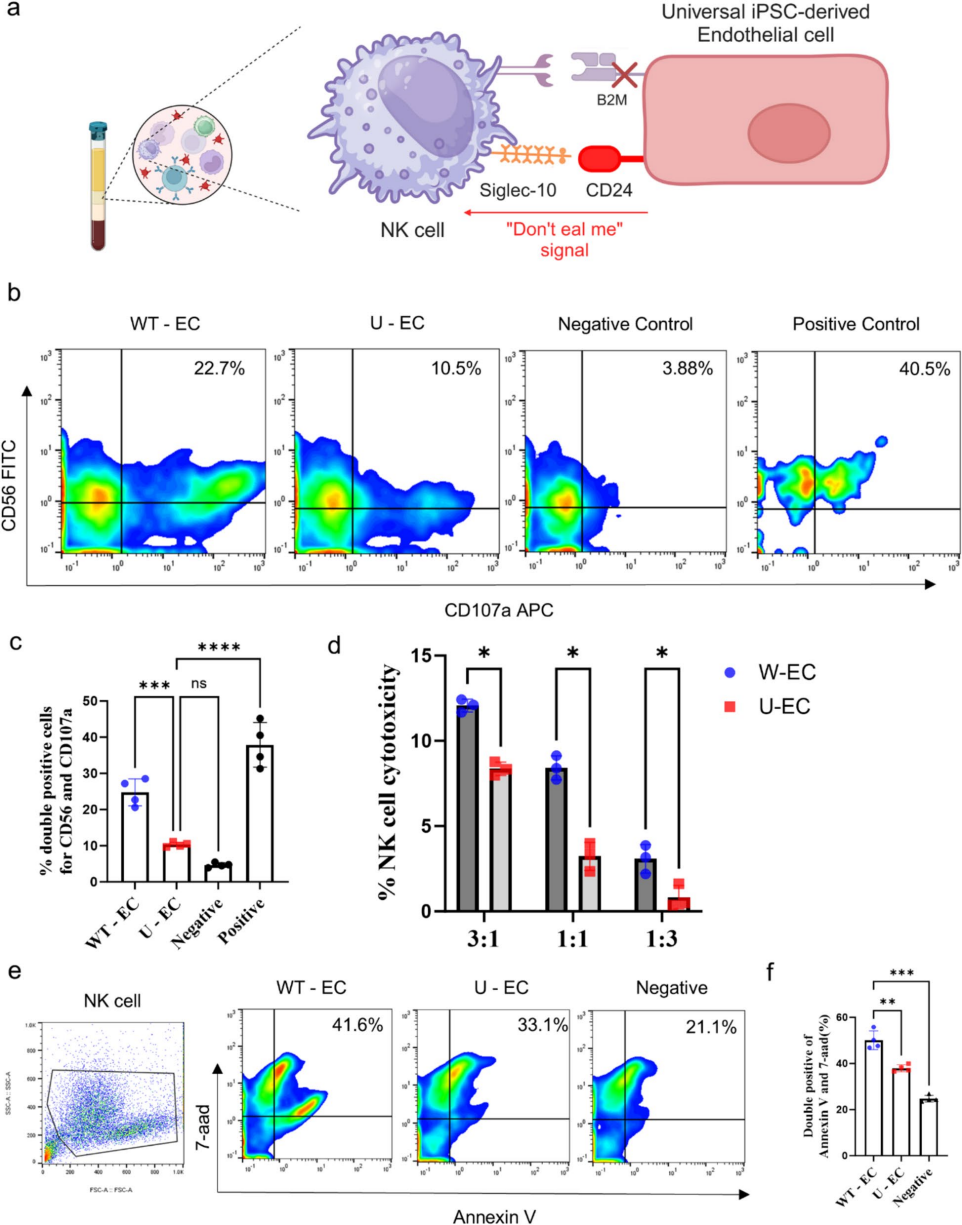
In vitro NK cell responses to iPSC-ECs. **a** Schematic diagram of the strategy to evade NK cell-mediated attack by overexpressing the'don’t eat me'signal CD24 in U-ECs **b** Representative flow cytometry analysis of CD107a on CD56^+^ NK cells: CD107a^+^ cells were used as an indicator to measure the degranulation of NK cells stimulated by ECs. NK cells treated with PMA/ionomycin served as positive control. **c** Quantification of percentage of double positive cells for CD56 and CD107a in flow cytometry analysis. Each group n = 4, mean ± SD, ***p < 0.001, ****p < 0.0001, ns; not statistically significant. **d** LDH release assay of NK cytotoxicity: histograms representing the percentage of NK cytotoxicity at the indicated effector/target (E/T) ratios. Each group n = 3, mean ± SD, *p < 0.05. **e** Representative flow cytometry analysis of apoptosis in ECs co-cultured with NK cells, using annexin V and 7-AAD double-positive expression. **f** Quantification of apoptotic cells. Each group n = 4, mean ± SD, **p < 0.01, ***p < 0.001. Statistical differences between the groups were determined by ordinary one-way ANOVA with post-hoc Tukey test

To verify whether the “don’t eat me signal” CD24 is sufficient to evade NK cell activity in co-culture system in vitro (Fig. 4A). First, we assessed the upregulation of degranulation marker CD107a (LAMP-1) of NK cells from UCB via MACS sorting (Fig. S3D). We found that NK cells reacted higher to WT-ECs (22.7%) than U-ECs (10.5%) (Fig. 4B and C). Secondly, we examined the Lactate Dehydrogenase (LDH) release assay to quantify the NK cell cytotoxicity to ECs via co-incubation. When NK cells attack and destroy target cells, it leads to damage to the cell membrane, causing intracellular LDH to be released into the extracellular space. We observed that NK cell cytotoxicity was significantly reduced when NK cells were incubated with U-ECs in different E/T ratios (Fig. 4D). Quantitative flow cytometry analysis showed that NK cells were more activated in the WT-EC group, leading to increased apoptosis (Fig. 4E). In conclusion, the over-expression of CD24 plays a significant role in evading NK cell-mediated lysis by functioning as a “don’t eat me” signal, thereby inhibiting NK cell activation.

### Establishment of a reconstituted human immune system in humanized mice

**Fig. 5:**
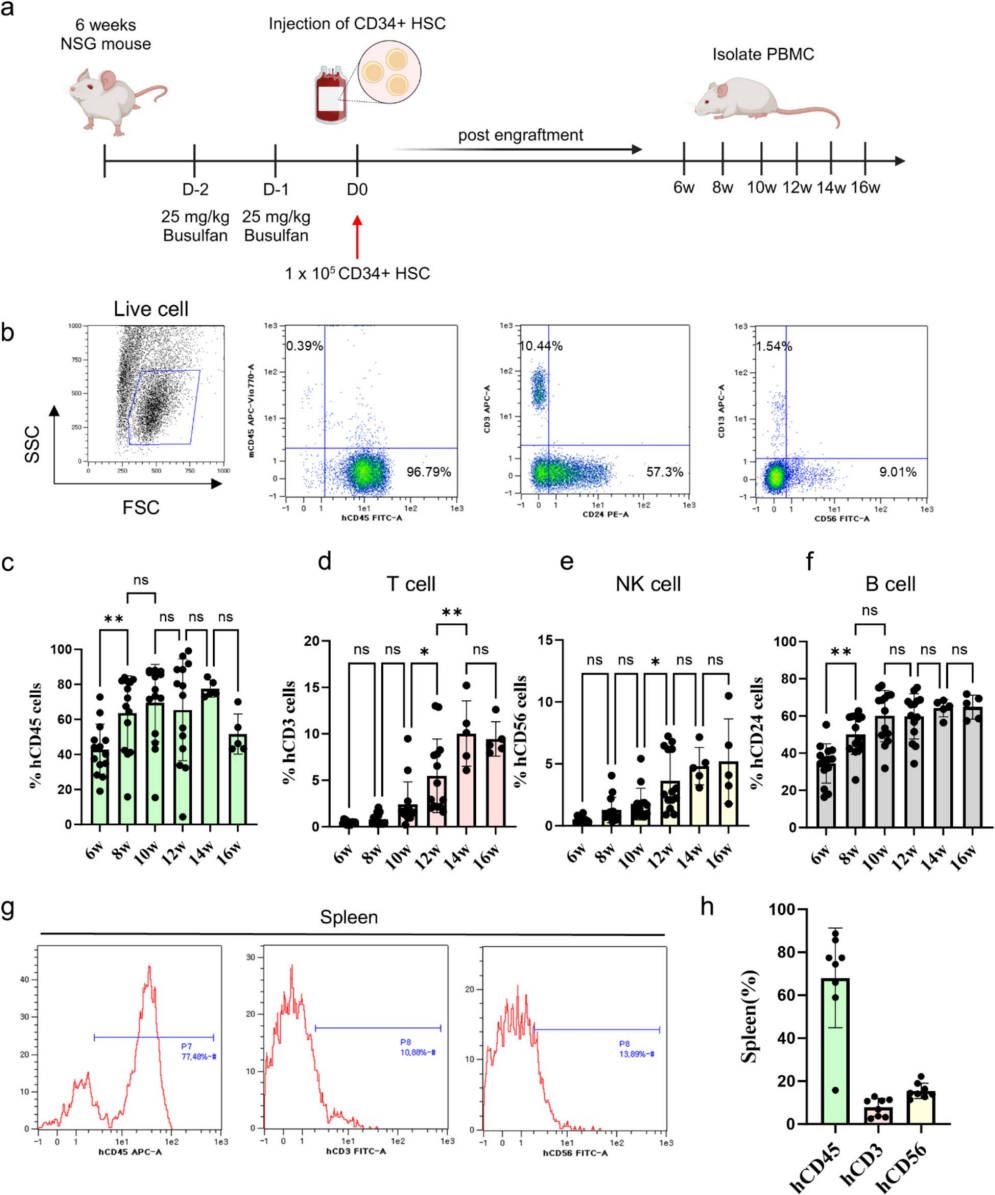
Reconstitution of humanized mice. **a** Schematic diagram of the strategy for generation of humanized mice. **b** Representative flow cytometry analysis of human peripheral blood mononuclear cells (PBMCs) in humanized mice at 12 weeks post-transplantation demonstrates readily detectable levels of various human immune cell types. **c**-**f** Kinetic analysis of the reconstitution of various human immune cell types (hCD45 +, T cells-hCD3 +, NK cells-hCD56 +, and B cells-hCD24 +) in humanized NSG mice. Peripheral blood was collected from the humanize mice at different time points post-transplantation (6 weeks: n = 14; 8 weeks: n = 14; 10 weeks: n = 14; 12 weeks: n = 14; 14 weeks: n = 5; 16 weeks: n = 5, mean ± SD, *p < 0.05, **p < 0.01, ns; not statistically significant). **g** Representative flow cytometry analysis of human CD45, human T (CD3) and NK (CD56) cells in the spleen of humanized mice at 12 weeks post-transplantation. **h** Quantification of human immune cells in spleen of humanized mice (n = 8). Statistical differences between the groups were determined by ordinary one-way ANOVA with post-hoc Tukey test

To establish an in vivo human immune response model in mice, we developed humanized mice following the schematic shown in Fig. 5A. For humanization, we injected busulfan into NSG mice twice for bone marrow depletion, followed by injecting human CD34^+^ HSCs isolated from human UCB. The distribution of human immune cells in the peripheral blood of NSG mice was analyzed by flow cytometry analysis every 2 weeks post-injection using the retro-orbital bleeding method. After 12 weeks post-injection, a significant percentage of human cells was identified by flow cytometry in the blood of NSG mice (Fig. 5B and C). Different human immune cell subtypes expressing hCD3 (T cells), hCD56 (NK cells), and hCD24 (B cells) were also evaluated. The ratio of human T cells and NK cells, the key mediators of HLA-driven immune responses, continuously increased by 12 weeks after humanized induction, at which point cell transplantation experiments were conducted (Fig. 5D-F). In addition, we analyzed the presence of human immune cells within the spleen through flow cytometry (Fig. 5G and H). Taken together, we confirmed that humanized mice with a human immune system were successfully generated from NSG mice.

### U-ECs demonstrated enhanced survival and immune evading capacity in humanized mice.

**Fig. 6:**
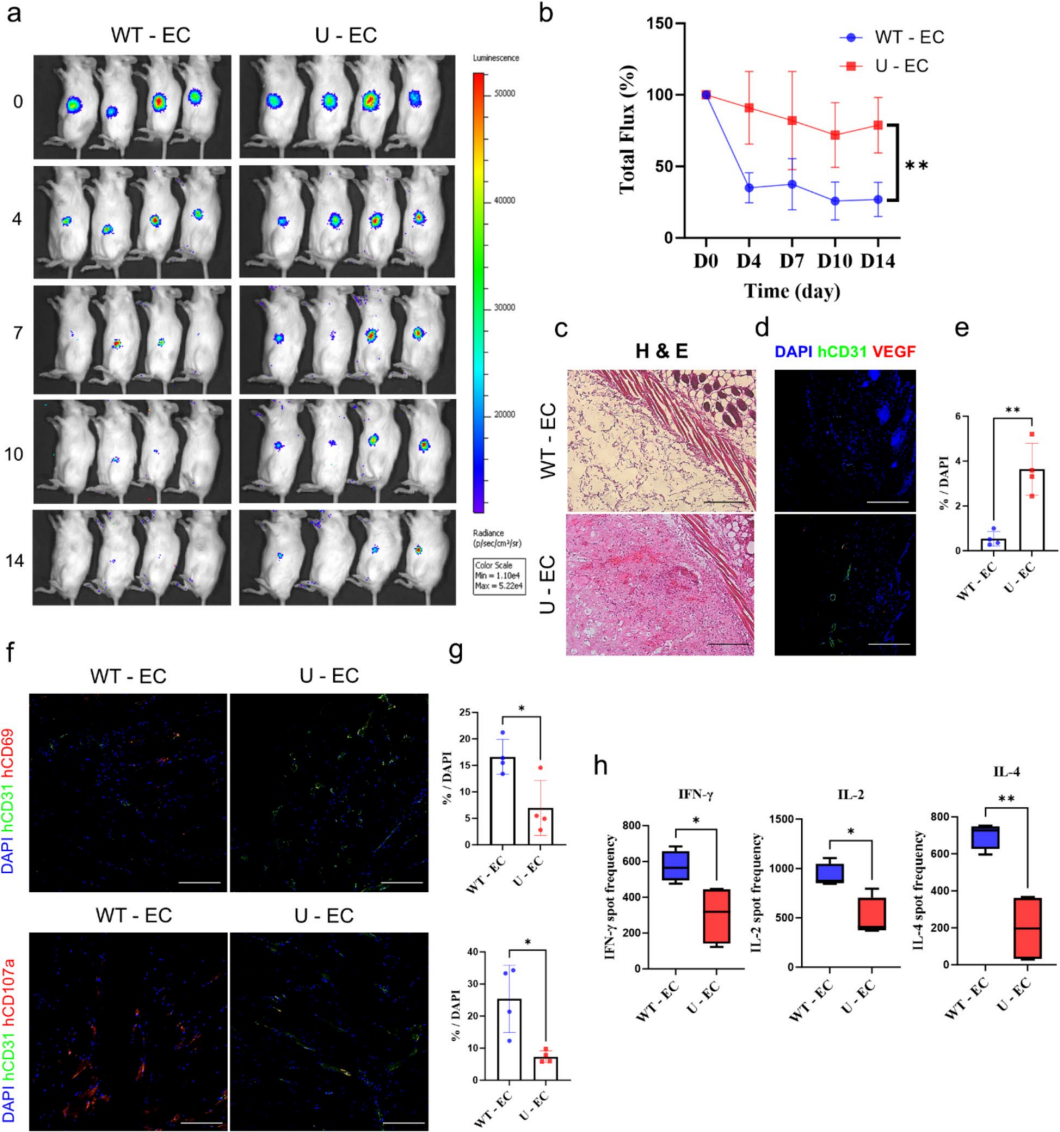
Survival of U-ECs in humanized mice. **a** Bioluminescence image of ECs monitored from day 0–14 after transplantation. **b** Total flux of ECs transplanted humanized mice monitored from day 0–14 after transplantation. n = 4, mean ± SD, **p < 0.01. Statistical differences between the groups were determined by two-way ANOVA with post-hoc Bonferroni’s test. **c** Representative histological (H&E) analysis of ECs transplanted humanized mice. **d** Representative confocal images of ECs (hCD31: green, VEGF: red, DAPI: blue). Scale bar, 100 µm. **e** Quantification of ECs normalized to hCD31^+^ and VEGF^+^ cells (n = 4) (f) Immunofluorescence analysis of the implants harvested on day 6 using immune activation (hCD69, hCD107a; red) markers, endothelial cell marker (hCD31; green), and DAPI (blue). Scale bar, 50 µm. **g** Quantification of detected T and NK cell activations normalized to hCD69^+^ and hCD107^+^ cells (*n* = 4). (h) IFN-γ, IL-2, and IL-4 Elispot assays were performed at days 6 post-transplantation. Each group n = 4, mean ± SD, *p < 0.05, **p < 0.01. Statistical differences between the groups were determined by unpaired student’s *t*-test

Based on in vitro data, we performed allogeneic transplant studies in humanized mice possessing human immune systems. Cell survival was quantitatively tracked by bioluminescent imaging system. All cell lines were transduced to express Luciferase by lentivirus to enable cell tracking, and their survival was measured based on signal intensity using an IVIS spectrum (Fig. 6A). As expected, WT-EC grafts in Matrigel plugs showed dramatic cell death, as the signal intensities decreased significantly 4 days after transplantation compared to U-EC group (WT-ECs = 35.07%, and U-ECs = 90.94%; Fig. 6B). By H&E staining, we observed that the U-ECs survived more effectively than WT-ECs in humanized mice (Fig. 6C). To determine the presence of ECs within the humanized microenvironment established, we performed immunohistochemistry on Matrigel-plugged tissues. Immunostaining confirmed that U-ECs were more prevalent and exhibited enhanced angiogenic functionality compared to WT-ECs (Fig. 6D and E). To confirm whether the transplanted endothelial cells were targeted and eliminated by human immune cells after the Matrigel plug, we fluorescently stained Matrigel-plugged tissue. The hCD69 and hCD107a immunostaining showed that human immune cells, specifically T cells and NK cells, were activated in response to the transplanted cells (Fig. 6F). Compared to the U-EC group, the WT-EC group showed increased activated immune cells (Fig. 6G). We next performed ELISpot assays using recipient splenocytes and donor cells to evaluate the cellular immune response by measuring cytokine production at the single-cell level. Recipients of WT-ECs induced strong IFN-γ, IL-2, and IL-4 responses, whereas recipients of U-ECs exhibited a significantly lower cellular IFN-γ, IL-2, and IL-4 response (Fig. 6H). These observations validated the immune evasion capability of U-ECs, previously identified in vitro, using a humanized mouse model and confirmed that U-ECs survived for a longer period in vivo compared to WT-ECs.

### Evaluation of the therapeutic potential of U-ECs for blood flow restoration in a humanized PAD mouse model

**Fig. 7:**
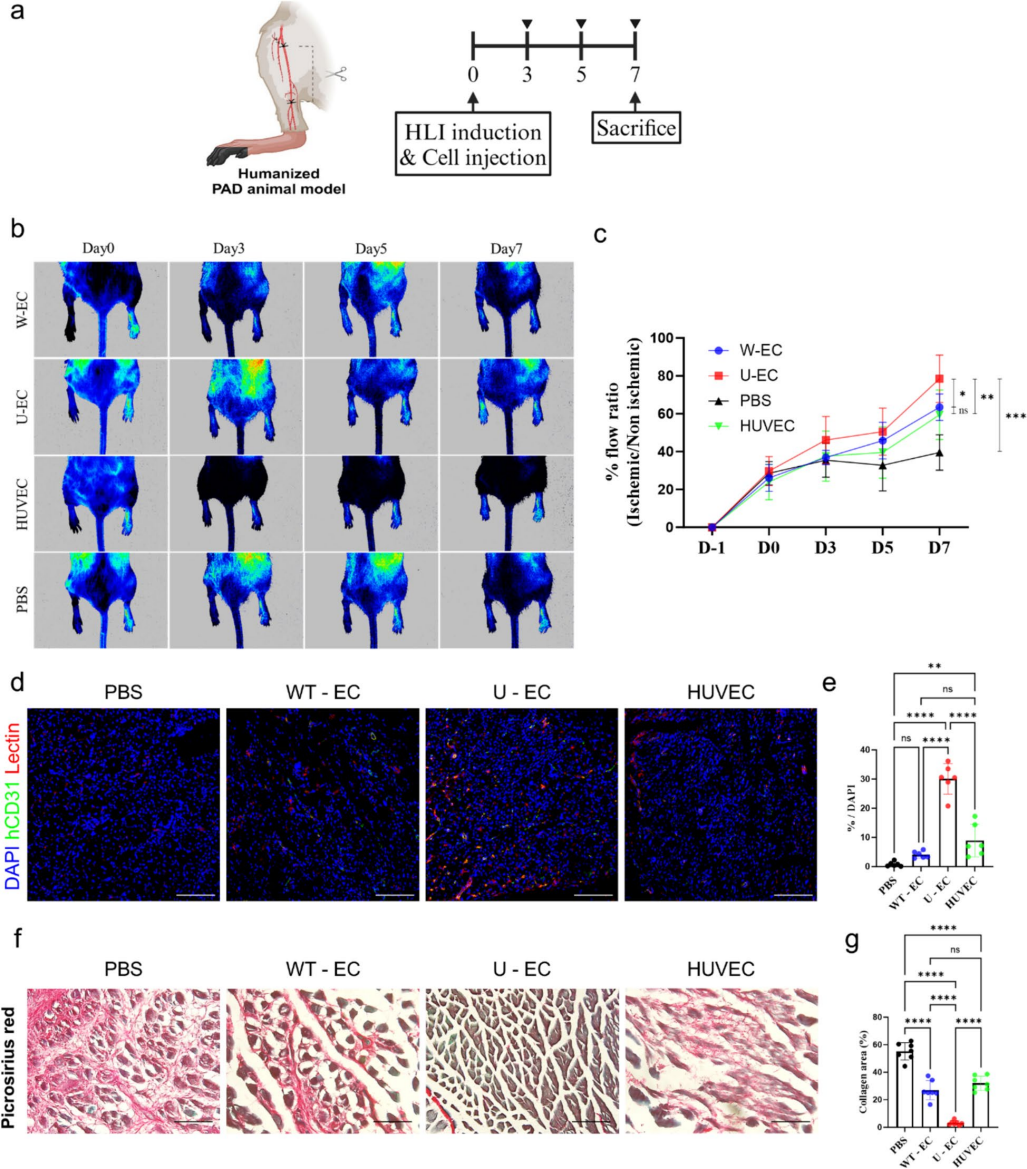
Therapeutic efficacy of ECs to improve blood flow during hindlimb ischemia in humanized mice after transplantation. **a** Schematic diagram illustrating the strategy for hindlimb ischemia induction of humanized mice. **b**-**c** Representative images and graph showing the improvement in blood flow in the ischemic regions of each mouse group after transplantation (WT-EC: n = 8; U-EC: n = 8; PBS: n = 5; HUVEC: n = 7, mean ± SD, *p < 0.05, **p < 0.01, ***p < 0.001, ns; not statistically significant). **d** Representative immunofluorescence images of engraftment and extent of angiogenesis of ECs (hCD31: green, Lectin: red, DAPI: blue). Scale bar, 50 µm. **e** Quantification of cells expressing hCD31 and Lectin in each group. Each group n = 6, mean ± SD, **p < 0.01, ****p < 0.0001, ns; not statistically significant. **f**-**g** Cryosections of muscle samples from hindlimb mice were stained with picrosirius red and muscle necrosis were measured. Scale bar = 100 μm. Each group n = 7, mean ± SD, ****p < 0.0001, ns; not statistically significant. Statistical differences between the groups were determined by two-way ANOVA with post-hoc Bonferroni’s test **c** and ordinary one-way ANOVA with post-hoc Tukey test **e**, **g**

Translational studies of iPSC-derived ECs have shown their potential for use in cellular therapies to treat various forms of ischemic diseases. To achieve a more accurate prediction, we evaluated the therapeutic efficacy of U-ECs by transplanting ECs into a humanized PAD mouse model with surgically induced hindlimb ischemia (Fig. 7A). In this PAD model, ischemia was induced by ligating the femoral artery. After surgery, vehicle groups (PBS), WT-ECs, U-ECs, and HUVECs (control) were injected at the ligation site. Therapeutic efficacy was assessed by evaluating the blood flow in the ischemic limbs using Laser Doppler Imaging on days 0, 3, 5, and 7 post-surgery (Fig. 7B). In the U-ECs transplanted group, a significant increase in flow ratio was observed on day 7 post-surgery (Fig. 7C). To estimate hindlimb functional recovery, the survival of transplanted endothelial cells and the formation of new microvessels were determined by analyzing hCD31 and Lectin (Fig. 7D and S4A). As expected, there was virtually no presence of hCD31 and Lectin-positive vessels in the PBS group, and only a small number of double-positive vessels were detected in the WT-ECs and HUVECs groups. Notably, a significantly improved density of double-positive vessels was observed in the U-EC group (Fig. 7E). We further assessed whether the transplanted U-ECs were found to integrate into the endothelial layer of pre-existing mouse vessels. Co-staining of human VE-Cadherin and mouse CD31 revealed co-localization, suggesting that the transplanted U-ECs successfully engrafted and integrated into the vasculature of the humanized PAD mouse model (Fig. S4B). In contrast to the pronounced angiogenesis in the ischemic muscle tissue, arteriogenic remodeling in the neurovascular bundles remained minimal, even in the U-EC group (Fig. S4C and S4D). Furthermore, to measure the alleviation of muscle degeneration, the ratio of non-muscular area to total area was quantified. Muscle fibrosis, caused by ischemic tissue damage, was evaluated using picrosirius red staining and quantification of the fibrotic area. The results showed that the degenerative region was largest in the PBS group at approximately 60%, whereas the WT-EC and HUVEC groups had similar levels of around 30%. In contrast, the U-EC group exhibited a significantly reduced degenerative region at around 3% (Fig. 7F and G). Collectively, U-EC transplantation significantly improved blood flow, microvessel formation, and reduced muscle degeneration. These results demonstrate that U-ECs have significant therapeutic potential for functional recovery in the humanized PAD mouse model.

### Assessment of immune response after the injection of U-ECs into the ischemic limb

**Fig. 8:**
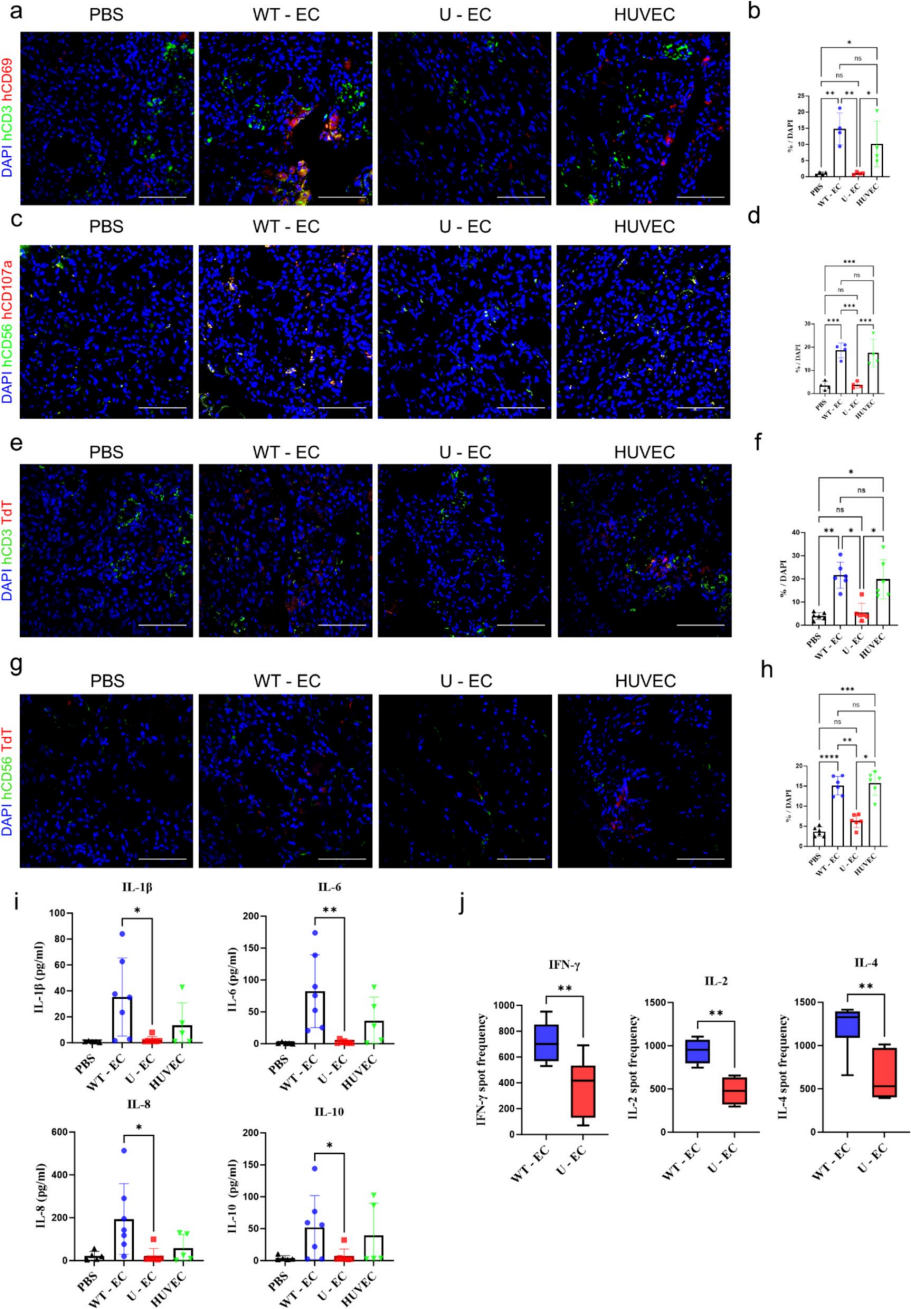
Assessment of immune evading post U-ECs administration in the hindlimb region. **a**-**d** Representative immunofluorescence images of the hindlimb region harvested on day 7 using immune activation (hCD69 and hCD107a; red) markers, immune cell marker (hCD3 and hCD56; green), and DAPI (blue). Scale bar, 50 µm. Quantification of cells expressing hCD3^+^/hCD69^+^ and hCD56/hCD107a in each group. Scale bar, 50 µm. Each group n = 4, mean ± SD, *p < 0.05, **p < 0.01, ***p < 0.001, ns; not statistically significant. (**e–h**) Representative confocal images of TUNEL assay. Each hindlimb region was stained with terminal deoxyribonucleotidyl transferase (TdT) for detection of DNA breaks and with DAPI for nuclei detection. Human T cells and NK cells (hCD3 and hCD56: green) were also imaged. Quantification of TdT-positive cells in randomized fields. Each group n = 6, mean ± SD, *p < 0.05, **p < 0.01, ***p < 0.001, ****p < 0.0001, ns; not statistically significant. (**i**) Serum collected from the humanized mice in each group on day 7 were subjected to cytometric bead array to analyze the levels of IL-1β, IL-6, IL-8, and IL-10. (PBS: *n* = 5; WT-EC: *n* = 7; U-EC: *n* = 7; HUVEC: n = 5, mean ± SD, *p < 0.05, **p < 0.01). (j) IFN-γ, IL-2, and IL-4 Elispot assays were performed at days 7 after hindlimb ischemia induction. Each group n = 7, mean ± SD, **p < 0.01. Statistical differences between the groups were determined by unpaired student’s *t*-test (**j**) and ordinary one-way ANOVA with post-hoc Tukey test (**b**, **d**, **f**, **h**, **i**)

To determine whether a human immune cell response occurred in each group, staining for immune cells and activation markers was performed. The staining results for the T cell marker hCD3 and the T cell activation marker hCD69 showed significantly less T cell activation in the U-EC group (Fig. 8A and B). Similarly, staining with the NK cell marker hCD56 and the NK cell activation marker hCD107a revealed that NK cell activation was significantly reduced in the U-EC group (Fig. 8C and D). Additionally, we performed a TUNEL assay to confirm whether U-ECs actually evaded the immune response in vivo, resulting in reduced apoptosis. Tunnel assays indicated that lower levels of apoptosis occurred within the U-EC group (Fig. 8E-H). Furthermore, we assessed the levels of inflammatory cytokines after U-EC transplantation using a human cytometric bead array (CBA) to determine the extent to which the cells trigger or suppress an inflammatory response. We measured that the levels of inflammatory cytokines, including IL- 1β, IL-6, IL-8, and IL-10, were the most elevated in the WT-EC group and significantly lower in both the U-EC and PBS groups (Fig. 8I). ELISpot assays demonstrated that allogeneic transplantation of WT-ECs resulted in a high systemic IFN-γ, IL-2, and IL-4 spot frequency in splenocytes, whereas U-ECs did not induce a humoral immune response (Fig. 8J). These results indicate that, compared to the WT-ECs and HUVECs groups, the U-EC group demonstrated a reduced immune response, which enabled more effective angiogenesis, leading to a significant therapeutic effect in the humanized PAD mouse model.

## Discussion

In this study, we successfully generated hypoimmunogenic iPSCs through targeted genome editing using the CRISPR-Cas9 system. The principal aim was to address the immunogenicity challenges that have historically limited the importance of iPSCs. By knocking out the B2M gene, which is crucial for HLA class I expression, and CIITA, the master regulator of HLA class II, we effectively reduced the immunogenicity of these cells. The generation of hypoimmunogenic iPSCs represents a strategic advancement in regenerative medicine, particularly in the realm of transplantation. HLA class I molecules typically present intracellular antigens to CD8 + cytotoxic T cells, while HLA class II molecules are involved in antigen presentation to CD4 + helper T cells. By knocking out B2M and CIITA, we effectively disrupted these pathways, thereby reducing immune recognition and rejection. By eliminating HLA expression, U-iPSCs can potentially evade both the adaptive immune response mediated by T cells and the innate immune response. However, the absence of HLA class I molecules could render these cells susceptible to NK cell-mediated lysis, a phenomenon known as “missing-self” recognition. The “missing-self” phenomenon was addressed by the overexpression of CD24, a “don’t eat me” signal. This strategic modification is vital for balancing immune evasion while preventing undue susceptibility to NK cell-mediated lysis.

We show that our human-engineered universal iPSCs maintained their pluripotency and could be efficiently differentiated into endothelial cells. The U-ECs expressed typical endothelial markers such as CD31 and CD144, indicating successful differentiation and functional endothelial identity. In addition, the angiogenesis assay demonstrated that the U-ECs had functional angiogenic abilities in vitro but not as good as the HUVEC control. This ability to generate hypoimmunogenic endothelial cells from U-iPSCs is a critical step forward in developing “off-the-shelf” cell therapies for vascular regeneration and tissue engineering.

Endothelial cells play a pivotal role in vascular health, and their potential use in therapeutic angiogenesis could revolutionize the treatment of ischemic diseases. However, the immunogenicity of transplanted cells is a critical hurdle in cell-based therapies, particularly when allogeneic cells are used. Here, we observed a markedly reduced stimulatory effect of U-ECs on allogeneic immune cells, as compared to WT-ECs. We found that U-ECs prevented the activation of CD8^+^ cytotoxic T cells and CD4^+^ helper T cells in vitro, which are key players in allograft rejection. This reduced stimulation is critical, as it suggests that U-ECs could be transplanted across HLA barriers with minimal risk of eliciting a robust T cell-mediated immune response. This could significantly broaden the applicability of these cells in a clinical setting, potentially allowing for universal donor cells that do not require HLA matching.

Despite the absence of HLA molecules, which typically serve as an inhibitory signal to NK cells, U-ECs demonstrated a reduced NK cell response. This was largely attributed to the over-expression of CD24, a “don’t eat me” signal, which interacts with Siglec-10 on NK cells by inhibiting their activation and cytotoxic response. The careful balance achieved between reducing T cell recognition and mitigating NK cell activation is a significant accomplishment in the field of hypoimmunogenic cell therapy. The use of CD24 as an immune checkpoint to mitigate NK cell activity represents a novel approach in the context of stem cell therapies. The CD24 over-expression successfully reduced NK cell degranulation and cytotoxic responses in vitro. As a “don’t eat me” signal to evade NK cell attacks, several studies have opted for the over-expression of CD47 [38–40]. In hypoimmunogenic cell therapy, the effectiveness of CD24 over-expression compared to CD47 remains a challenge. In general, the phagocytosis activity depends on the balance between prophagocytic (e.g. calreticulin, Fc-mediated opsonization) and anti-phagocytic surface signals (e.g. CD47, CD24) [41]. Aroldi et al. reported that when the blockade of CD24 and CD47 signals occurred individually, the phagocytic rate was similarly enhanced [42]. Considering the functional diversity of anti-phagocytic surface signals, our arguments need to be further validated by comparing the NK cell evasion capabilities of CD24 and CD47 in iPSCs.

To assess the in vivo immunogenicity and therapeutic efficacy of U-ECs, we established a humanized mouse model bearing physiologically relevant human immune system as an in vivo tool by engrafting human CD34^+^ HSCs into immunodeficient mice. Due to variability in the human immune system reconstitution among individual mice, we employed at least 4 animals per experimental group, as supported by previously established protocols using humanized mouse models [43, 44]. After cell transplantation, the survival of U-ECs in the humanized mouse model was significantly longer than that of WT-ECs, which were subject to rapid immune rejection. The extended survival and improved engraftment of U-ECs in this model underscore the success of our immune-evasion strategy. This enhanced survival underscores the effectiveness of our CRISPR-Cas9 modifications in reducing immune recognition and rejection. The in vivo results corroborate our in vitro findings, highlighting the potential of U-ECs for use in regenerative therapies without the need for immunosuppression.

Finally, we assessed the therapeutic efficacy of U-ECs in a humanized PAD mouse model. U-ECs demonstrated a significant ability to restore blood flow in ischemic limbs, which is a critical therapeutic goal in PAD treatment. The U-EC-treated groups significantly enhanced blood flow recovery and exhibited robust angiogenic capacity, as evidenced by increased capillary density and reduced tissue fibrosis compared to WT-ECs and vehicle control groups. These results are promising, demonstrating that U-ECs not only survive in an immune-competent environment but also retain their therapeutic functionality, making them a promising candidate for treating vascular diseases.

In this study, we primarily focused on angiogenesis-related parameters, such as capillary formation. Given that arteriogenesis plays a critical role in restoring blood flow through the remodeling of pre-existing collateral arteries [45], we also examined arteriogenic remodeling in the neurovascular bundles, which was scarcely detectable even in the U-EC group. This limited arteriogenic response may be attributed to two key factors: first, the hindlimb ischemia in our model was induced by complete resection of the femoral artery, a method known to promote diffuse ischemia and favor angiogenesis over arteriogenesis [46]. Second, tissue sampling was performed at 7 days post-transplantation, a time point at which angiogenic activity typically precedes the onset of substantial arteriogenic remodeling [47]. Therefore, the observed pro-angiogenic effects in our data are consistent with this surgical context and with the 7-day post-injury time point. Nonetheless, further studies utilizing chronic ischemia models that better mimic the progressive nature of human PAD are warranted to more comprehensively evaluate the arteriogenic potential of U-ECs and their long-term therapeutic efficacy.

The generation of human-engineered universal iPSC-derived endothelial cells using CRISPR-Cas9 represents a significant step forward in the field of regenerative medicine. These cells offer a potential solution to the problem of immune rejection in cell-based therapies. However, further studies are needed to evaluate the long-term safety and efficacy of these cells in different disease models and to explore the potential for scaling up their production for clinical applications. Future studies should also explore the integration of additional genetic modifications to further enhance the therapeutic efficacy of U-ECs, such as the introduction of pro-angiogenic factors or enhanced homing capabilities. Additionally, the impact of CD24 over-expression on other immune functions and the potential for unintended off-target effects of CRISPR-Cas9 editing warrant careful investigation.

## Conclusions

In summary, our study demonstrates the feasibility of creating hypoimmunogenic iPSCs and their derivatives that can evade immune detection and function effectively in vitro. Furthermore, we applied the PAD model in humanized mice and validated its therapeutic efficacy in vivo. This approach holds great promise for the future of regenerative therapies through overcoming the barriers posed by immune rejection, offering new avenues for the development of universal and “off-the-shelf” cell therapies.

## Supplementary Information

Supplementary material 1.

## Data Availability

All datasets generated or analyzed during the current study are included in this published article and its Supplementary Information. All additional files are included in the manuscript. The sequencing data supporting the results reported in this study have been deposited in the NCBI Sequence Read Archive under the accession number PRJNA1219490. Additional experimental details and more detailed data used or analyzed in this study are available from the corresponding author upon reasonable request.

## Abbreviations

AcLDL: Acetylated low-density lipoprotein
B2M: Beta2 microglobulin
CBA: Cytometric bead array
CIITA: Class II transactivator
DNB: DNA nanoball
ECs: Endothelial cells
EGM-2: Endothelial growth medium-2
ELISA: Enzyme linked immunosorbent assay
FBS: Fetal bovine serum
HLA: Human leukocyte antigens
hUCB: Human umbilical cord blood
HUVECs: Human umbilical vein endothelial cells
IGV: Integrative Genomics Viewer
iPSCs: Induced pluripotent stem cells
IRB: Institutional Review Board
IVIS: In vivo imaging system
MACS: Magnetic-activated cell sorting
MFI: Mean fluorescence intensity
MHCs: Major histocompatibility complexes
MNCs: Mononuclear cells
mAb: Monoclonal antibody
NK: Natural killer
PAD: Peripheral arterial disease
qRT-PCR: Quantitative real-time polymerase chain reaction
SD: Standard deviation
Siglec-10: Sialic-acid-binding Ig-like lectin 10
TAMs: Tumor-associated macrophages
TME: Tumor microenvironment
U-ECs: Universal iPSC-derived ECs
U-iPSCs: Universal iPSCs
VEGF: Vascular endothelial growth factor
WT-ECs: Wild-type iPSC-derived endothelial cells
WGS: Whole genome sequencing
