# Supplementary material for: Therapeutic effects of hypoimmunogenic universal human iPSC-derived endothelial cells in a humanized mouse model of peripheral artery disease
Source: Stem Cell Res Ther. 2025 Aug 6;16:430. doi: 10.1186/s13287-025-04554-5 (PMC12330048; doi:10.1186/s13287-025-04554-5)
Supplement: Supplementary file 1 — Supplementary material 1. [file 13287_2025_4554_MOESM1_ESM.docx]

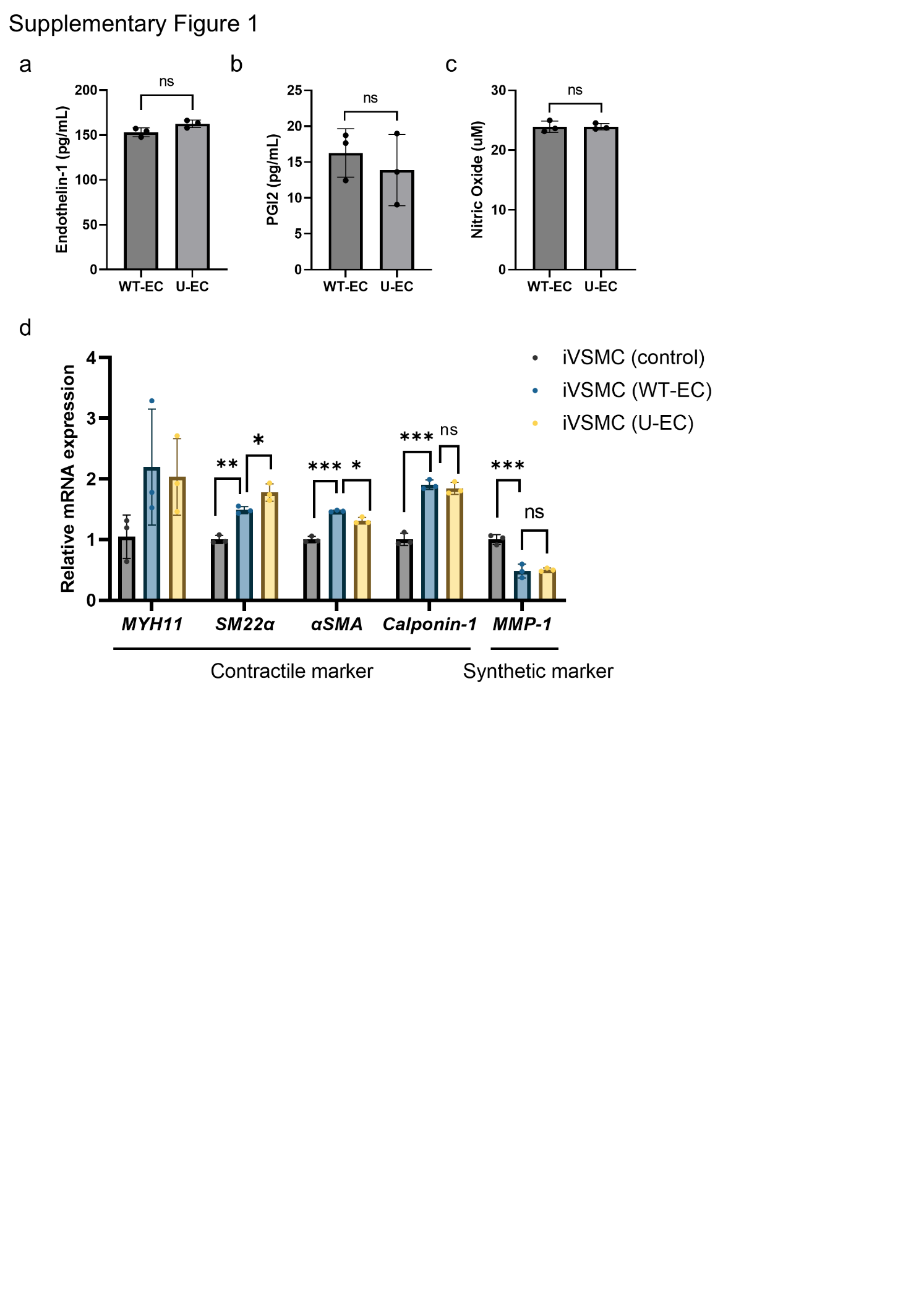


Supplementary Fig. 1. Secretion of vasoactive mediators from differentiated ECs and their impacts on iVSMCs.

(a-c) The amount of secreted endothelin-1 (a), PGI2 (b), and nitric oxide (c) from WT-ECs and U-ECs after 6-hour stimulation with 100 ng/mL of LPS was quantified by ELISA. (d) qRT-PCR analysis of the contractile marker genes, such as *MYH11, SM22α, αSMA, Calponin-1*, and gene associated with synthetic phenotype, *MMP-1*, in the VSMCs differentiated from iPSCs. VSMCs were cultured in the standard medium (control), or the conditioned medium harvested from WT-ECs and U-ECs, respectively. Each group n = 3, mean ± SD, *p < 0.05, **p < 0.01, ***p < 0.001, ns: not statistically significant. Statistical differences between the groups were determined by unpaired student’s *t*-test (a, b, c) and ordinary one-way ANOVA with post-hoc Tukey test (d).


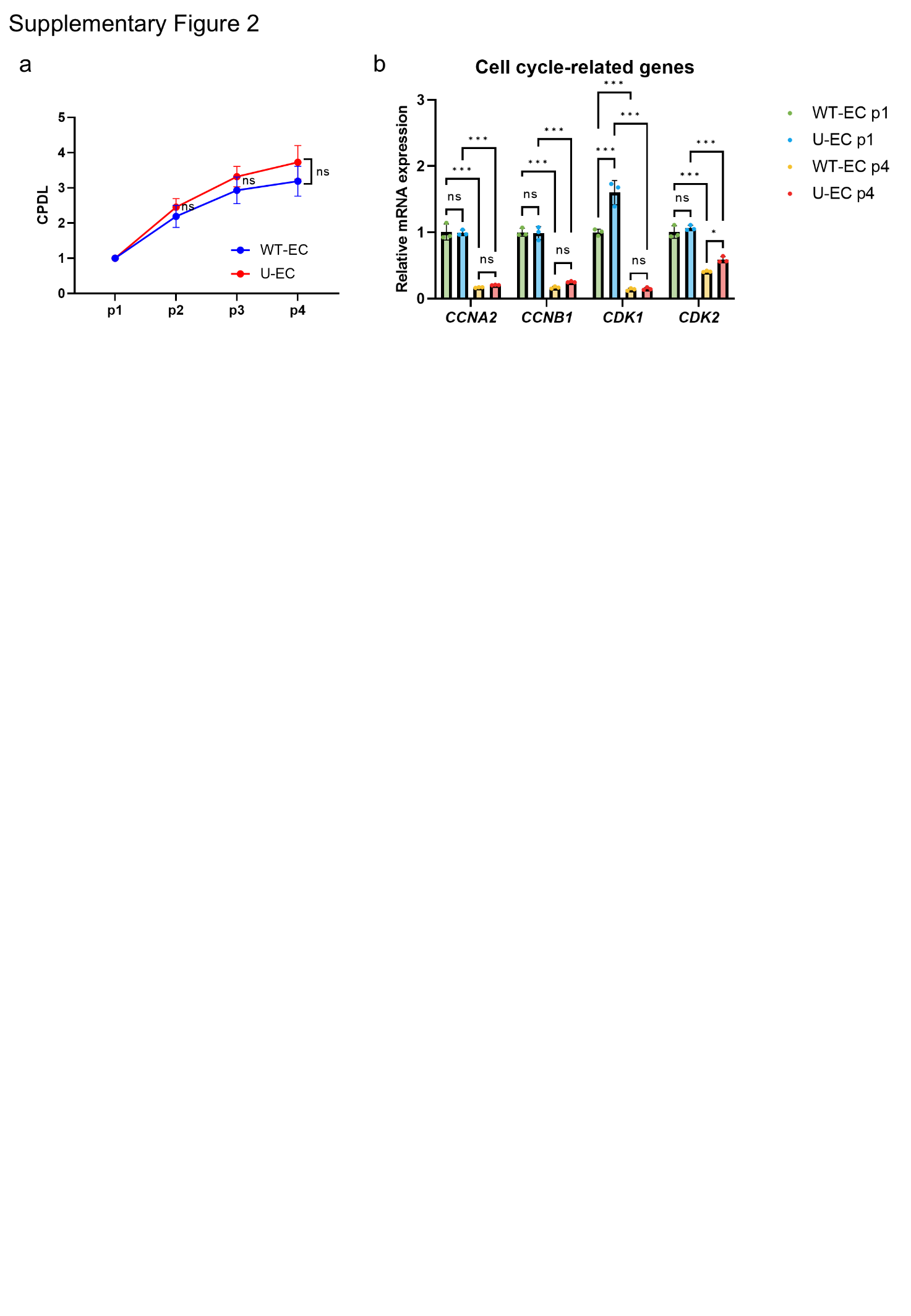


Supplementary Fig. 2. Proliferative capacity of differentiated ECs.

(a) CPDL of WT-ECs (blue) and U-ECs (red) across passages 1 to 4 (p1-p4). At each passage, population doubling level (PDL) was calculated using the formula: log_e_ (final cell number / initial seeding cell number). (b) qRT-PCR analysis of cell cycle-regulating genes, including *CCNA2, CCNB1, CDK1, and CDK2,* in the WT-ECs and U-ECs at passages 1 and 4. Note that no statistically significant differences were observed between WT-ECs and U-ECs at the corresponding passages. Each group n = 3, mean ± SD, ***p < 0.001, ns: not statistically significant. Statistical differences between the groups were determined by unpaired student’s *t*-test (a) and ordinary one-way ANOVA with post-hoc Tukey test (b).


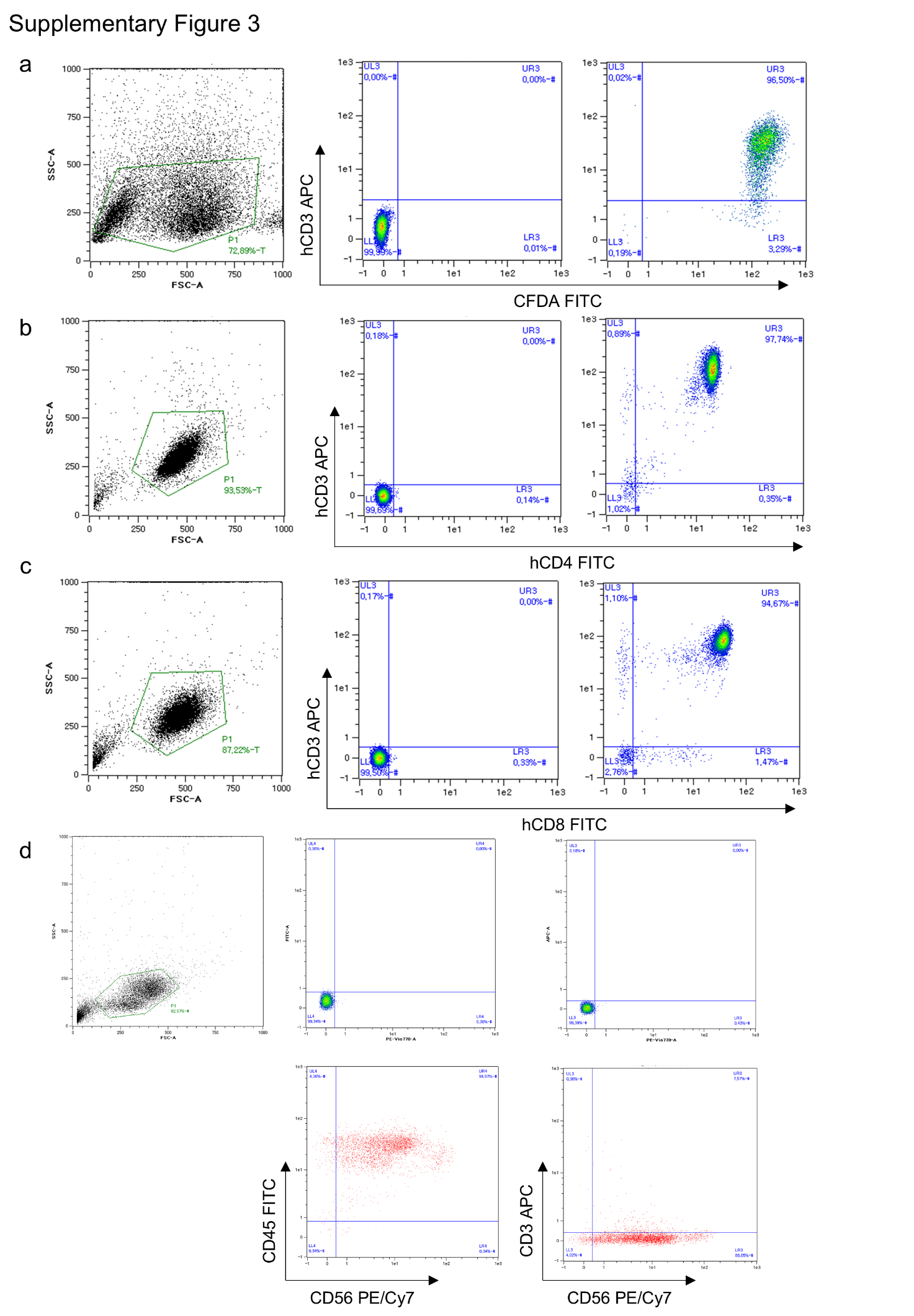


Supplementary Fig. 3. Isolation of immune cells

(a) Representative flow cytometry analysis of human CD3^+^ T cells, showing successful isolation from hUCB and effective CFDA staining for proliferation assessment. (b) Representative flow cytometry analysis to confirm isolation of CD4^+^ T cells from hUCB , with CD4^+^ cells gated among the CD3^+^ T cell population. (c) Representative flow cytometry analysis to confirm isolation of CD8^+^ T cells from hUCB, with CD8^+^ cells gated among the CD3^+^ T cell population. (d) Representative flow cytometry analysis of human CD56^+^ CD3^-^NK cells, gated using CD56 PE/Cy7 and CD45 FITC, distinguishing them from CD3^+^ T cells.


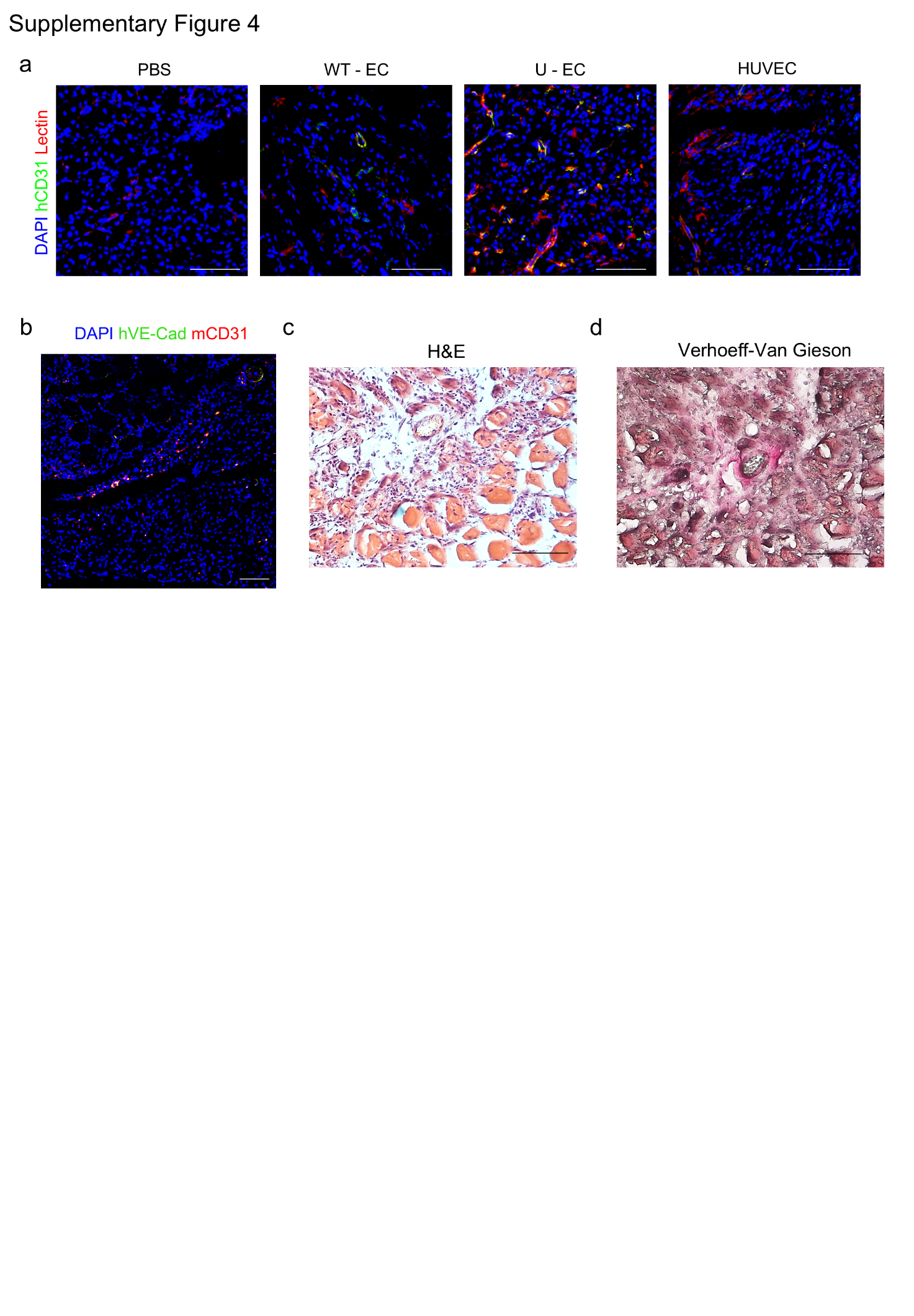


Supplementary Fig. 4. Histological analysis in the hindlimb ischemia model

(a) Representative magnified immunofluorescence images of Figure 7D. Note the engraftment of transplanted U-ECs and formation of vascular structure in the host mice (hCD31: green, Lectin: red, DAPI: blue). Scale bar, 50 µm. (b) Representative immunofluorescence image showing the integration of transplanted U-ECs into the endothelial layer of pre-existing mouse vessels. (hVE-Cad: green, mCD31: red, DAPI: blue). Scale bar, 50 µm. (c) Representative H&E analysis of the resected area in U-EC group. Scale bar: 50 µm. (d) Representative Verhoeff–Van Gieson staining of ischemic tissue in U-EC group. Scale bar: 50 µm.

Supplementary table 1. The list of primers used for qRT-PCR analysis

| human *GAPDH* | Forward | 5'- GTC AGT GGT GGA CCT GAC CT - 3’ |
| --- | --- | --- |
|  | Reverse | 5'- TGC TGT AGC CAA ATT CGT TG - 3’ |
| human *B2M* | Forward | 5'- TCT GGG TTT CAT CCA TCC GAC - 3’ |
|  | Reverse | 5’- CAC ACG GCA GGC ATA CTC AT - 3’ |
| human *CIITA* | Forward | 5’- ACC TCC CGA GCA AAC ATG AC - 3’ |
|  | Reverse | 5’- CCA GAT CCA CCT CCA CTA GGA T - 3’ |
| human *CD24* | Forward | 5’- GGG CTC GAG GGC CGT TC - 3’ |
|  | Reverse | 5’- CAG CAC GCA AGG GAA TGG AAA - 3’ |
| human *MYH11* | Forward | 5’- GGA GGA TGA GAT CCT GGT CA - 3’ |
|  | Reverse | 5’ - TTA GCC GCA CTT CCA GTT CT - 3’ |
| human *SM22α* | Forward | 5’ - CAA GCT GGT GAA CAG CCT GTA C - 3’ |
|  | Reverse | 5’ - GAC CAT GGA GGG TGG GTT CT - 3’ |
| human *αSMA* | Forward | 5’ - CAA GTG ATC ACC ATC GGA AAT G - 3’ |
|  | Reverse | 5’ - GAC TCC ATC CCG ATG AAG GA - 3’ |
| human *Calponin-1* | Forward | 5’ - TGA AGC CCC ACG ACA TTT TT - 3’ |
|  | Reverse | 5’ - GGG TGG ACT GCA CCT GTG TA - 3’ |
| human *MMP-1* | Forward | 5’ - CTG GGC CAC TAT TTC TCC GCT - 3’ |
|  | Reverse | 5’ - TCT CAC AGC TTC CCA GCG AC - 3’ |
| human *CCNA2* | Forward | 5’ - TGT CAG CTA TGA GTA AGA CTG GC - 3’ |
|  | Reverse | 5’ - CTG GTT TTA CTC TCA TCT TGC CAC - 3’ |
| human *CCNB1* | Forward | 5’ - ATG TGC CCC TGC AGA AGA AG - 3’ |
|  | Reverse | 5’ - TTT CCA GTG ACT TCC CGA CC - 3’ |
| human *CDK1* | Forward | 5’ - GGA AAT TGA GCG GAG AGC GA - 3’ |
|  | Reverse | 5’ - CAT GGC TAC CAC TTG ACC TGT A - 3’ |
| human *CDK2* | Forward | 5’ - CCA GTA CTG CCA TCC GAG AG - 3’ |

Supplementary table 2. The list of antibodies used in this study

| Name | Source | Catalog | Dilution |
| --- | --- | --- | --- |
| Anti-OCT4 | Santa Cruz Biotechnology | Cat# sc-5279 | 1:100 |
| Anti-SOX2 | Abcam | Cat# ab97959 | 1:100 |
| Rabbit Anti-CD31 | Abcam | Cat# ab28364 | 1:100 |
| Mouse Anti-Human vWF | BD | Cat# 555849 | 1:100 |
| Anti-PECAM1 antibody | Sigma Aldrich | Cat# CBL1337 | 1:100 |
| Anti-VECadherin antibody | abcam | Cat# ab7047 | 1:50 |
| Mouse Anti-VEGF | Santa Cruz Biotechnology | Cat# sc-7269 | 1:100 |
| Mouse Anti-CD69 | BD | Cat# 555531 | 1:50 |
| Mouse Anti-CD107a | BD | Cat# 560948 | 1:40 |
| Mouse Anti-Lectin | Vector Laboratories | Cat# RL-1102-2 | 1:50 |
| PE Mouse anti-Human TRA-1-81 | BD | Cat# 560161 | 1:100 |
| Human/Mouse SSEA-4 APC-conjugated | R&D | Cat# FAB1435A | 1:20 |
| PE Mouse Anti-Human CD31 | BD | Cat# 555446 | 1:50 |
| Alexa Fluor® 647 Mouse Anti-Human CD144 | BD | Cat# 561567 | 1:50 |
| APC anti-human CD3 Antibody | Biolegend | Cat# 344812 | 1:100 |
| FITC Mouse Anti-Human CD4 | BD | Cat# 555346 | 1:100 |
| FITC Mouse Anti-Human CD8 | BD | Cat# 555366 | 1:100 |
| FITC Mouse Anti-Human HLA-DR | BD | Cat# 555811 | 1:100 |
| FITC Mouse anti-Human CD56 (NCAM-1) | BD | Cat# 562794 | 1:50 |
| APC Mouse Anti-Human CD107a | BD | Cat# 560664 | 1:50 |
| FITC Mouse Anti-Human CD45 | BD | Cat# 555482 | 1:100 |
| PE Mouse Anti-Human CD24 | BD | Cat# 555428 | 1:100 |
| APC Mouse Anti-Human CD13 | BD | Cat# 561698 | 1:100 |
